# Histone lactylation drives oncogenesis by facilitating m^6^A reader protein YTHDF2 expression in ocular melanoma

**DOI:** 10.1186/s13059-021-02308-z

**Published:** 2021-03-16

**Authors:** Jie Yu, Peiwei Chai, Minyue Xie, Shengfang Ge, Jing Ruan, Xianqun Fan, Renbing Jia

**Affiliations:** Department of Ophthalmology, Shanghai Key Laboratory of Orbital Diseases and Ocular Oncology, Shanghai Ninth People’s Hospital, Shanghai JiaoTong University School of Medicine, Shanghai, People’s Republic of China

**Keywords:** Histone lactylation, Transcriptional activation, YTHDF2, m^6^A, Ocular melanoma

## Abstract

**Background:**

Histone lactylation, a metabolic stress-related histone modification, plays an important role in the regulation of gene expression during M1 macrophage polarization. However, the role of histone lactylation in tumorigenesis remains unclear.

**Results:**

Here, we show histone lactylation is elevated in tumors and is associated with poor prognosis of ocular melanoma. Target correction of aberrant histone lactylation triggers therapeutic efficacy both in vitro and in vivo. Mechanistically, histone lactylation contributes to tumorigenesis by facilitating YTHDF2 expression. Moreover, YTHDF2 recognizes the m6A modified PER1 and TP53 mRNAs and promotes their degradation, which accelerates tumorigenesis of ocular melanoma.

**Conclusion:**

We reveal the oncogenic role of histone lactylation, thereby providing novel therapeutic targets for ocular melanoma therapy. We also bridge histone modifications with RNA modifications, which provides novel understanding of epigenetic regulation in tumorigenesis.

**Supplementary Information:**

The online version contains supplementary material available at 10.1186/s13059-021-02308-z.

## Introduction

Posttranslational modifications of histones play vital roles in the maintenance of homeostasis by regulating DNA-dependent processes, including transcription, replication, and DNA repair, and these modifications function by changing the contact between nucleosomes or by recruiting nonhistone proteins [1, 2]. H4K20me2 and H4K20me3 are crucial for DNA replication initiation, which facilitates the recruitment of the Orc complex to replication origins [3]. ATXR5- and ATXR6-mediated H3K27me1 at specific genomic locations prevents DNA replication from occurring more than once per cell cycle [4]. In addition, phosphorylation of H2AX is one of the earliest reactions to DNA damage, and it is important for promoting efficient repair by recruiting repair machinery to damage sites [5]. Hence, there is a strong relationship between histone modifications and biological processes, and the functional significance of histone modifications has always been a research hotspot.

The dysregulation of histone modifications can shift the balance of transcription activation and repression, thus resulting in disease occurrence and progression [6]. For example, KDM4A-mediated H3K9me3 demethylation in oocytes is essential for proper zygotic genome activation and preimplant development after fertilization, while loss of KDM4A causes insufficient transcriptional activation of genes [7]. In addition, a histone acetylome-wide association study identified 4162 different H3K27ac peaks enriched in disease-related biological pathways between Alzheimer’s disease cases and controls [8]. In mixed-lineage leukemia (MLL), ASH1L-mediated H3K36me2 is associated with transcriptional activation and leukemic transformation [9]. Therefore, histone modifications are versatile marks closely related to disease onset and development, and the exploration of the role of histone modifications in the pathogenesis of diseases, especially tumorigenesis, has attracted increasing attention.

Histones can be modified in many ways, including acetylation, methylation, phosphorylation, ubiquitination, and SUMOylation, which have been known for a long time [1]. With the development of high-sensitivity mass spectrometry, various histone acylation marks derived from cellular metabolites were newly discovered, such as propionylation, butyrylation, succinylation, and malonylation [10]. More recently, Zhang et al. identified lactate-derived lactylation of histones as a newly discovered epigenetic modification that activates gene transcription. Intriguingly, lactate was produced by glycolysis under hypoxic conditions or during bacterial challenge, stimulating histone lactylation and subsequently activating downstream gene expression [11]. Furthermore, histone lactylation functions as a vital epigenetic regulator during pathogenesis. For example, elevated histone lactylation at reparative macrophage gene loci was found to promote the transition of macrophage from inflammatory to reparatory state in response to microbial ligands and various harmful cues [12]. In addition, Glis1-induced histone lactylation at pluripotency gene loci facilitated somatic cell reprogramming [13]. However, the role of histone lactylation in tumorigenesis remains unclear. Since Warburg effect (aerobic glycolysis) is one of the hallmarks of cancer [14], cancer cells tend to use ‘ferment’ glucose into lactate for energy generation even in aerobic conditions, with more lactate metabolized by glucose in a given time than normal cells [15]. Thus, histone lactylation in tumors is very likely to be aberrant and exploring the potential function of histone lactylation in tumorigenesis is interesting.

In this study, we identified, for the first time, that histone lactylation levels were upregulated in ocular melanoma. Inhibition of histone lactylation efficiently suppressed tumor progression. Mechanistically, histone lactylation promoted the transcription of YTH N6-methyladenosine RNA-binding protein 2 (YTHDF2), which recognizes the m^6^A modification site on the RNA of two tumor suppressor genes, PER1 and TP53, and promoted their degradation. Therefore, our data revealed a novel mechanism of histone lactylation in tumorigenesis and, for the first time, demonstrated the crosstalk of histone lactylation and m6A methylation in ocular melanoma.

## Results

### Elevated histone lactylation levels are associated with unfavorable prognosis for patients with ocular melanoma

**Fig. 1:**
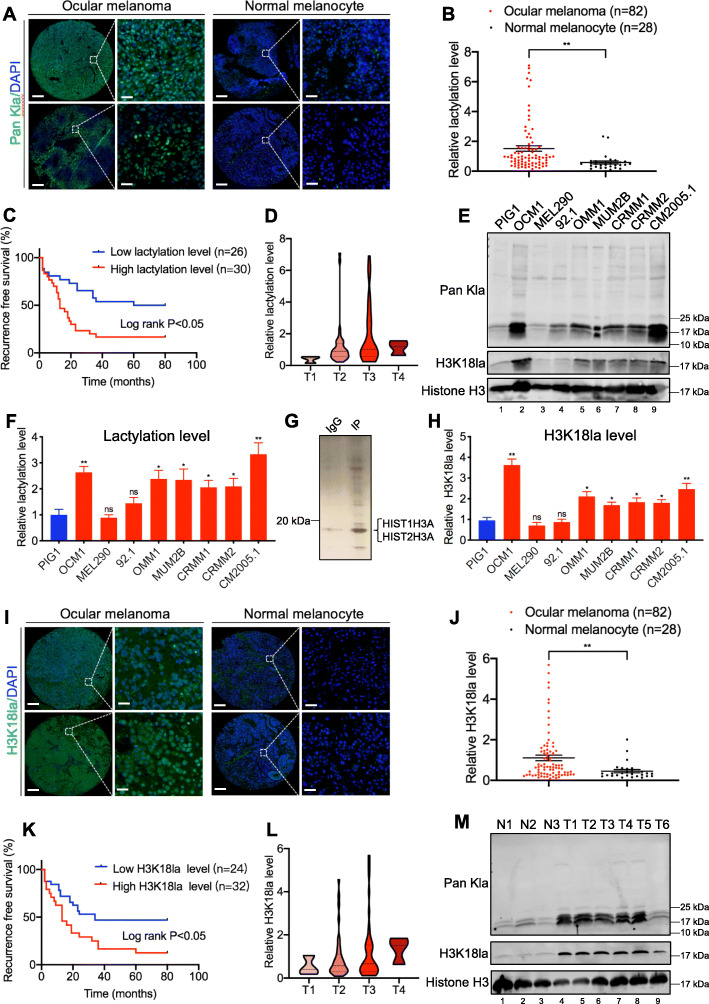
Ocular melanoma exhibited increased lactyation levels and associated with poor survival in UVM patients. **a, b** Lactyation levels in normal and tumor tissues. **a** Lactyation levels were visualized by immunofluorescence staining in normal and tumor tissues. Scale bar: left panel, 100 μm; right panel, 20 μm. **b** Statistical results of lactyation levels in normal and tumor tissues. Unpaired *t* test, ** *p* < 0.01. **c** Kaplan-Meier curves of time for recurrence showing the difference between ocular melanoma patients with low and high lactyation levels. “High lactylation” was defined as relative lactylation level of the samples (determined by luminance intensity) higher than 0.87, while those lower than 0.87 as “low lactylation”. *n* = 56, log-rank test, *p* < 0.05. **d** Lactyation levels in patients at AJCC stages T1 to T4. There were 3 ocular melanoma samples in T1 stage, 36 in T2 stage, 40 in T3 stage, and 3 in T4 stage. **e** Lactyation and H3K18la levels were detected in normal melanocytes and ocular melanoma cell lines by Western blot. **f** Densitometric analysis was performed to quantify and statistically compare lactyation levels in normal melanocytes and ocular melanoma cell lines that normalized to histone H3. *p < 0.05, **p < 0.01. **g** Silver staining-mass spectrometry (MS) of lactylated proteins. **h** Densitometric analysis was performed to quantify and statistically compare H3K18la levels in normal melanocytes and ocular melanoma cell lines that normalized to histone H3. *p < 0.05, **p < 0.01. **i, j** H3K18la levels in normal and tumor tissues. **i** H3K18la levels were visualized by immunofluorescence staining. Scale bar: left panel, 100 μm; right panel, 20 μm. **j**. Statistical results of H3K18la levels in normal and tumor tissues. Unpaired t test, **p < 0.01. **k** Kaplan-Meier curves of time for recurrence showing the difference between ocular melanoma patients with low and high H3K18la levels. “High H3K18la” was defined as relative lactylation level of the samples (determined by luminance intensity) higher than 0.53, while those lower than 0.53 as “low H3K18la”. *n* = 56, log-rank test, *p* < 0.05. **l** H3K18la levels in patients at AJCC stages T1 to T4. There were 3 ocular melanoma samples in T1 stage, 35 in T2 stage, 41 in T3 stage, and 3 in T4 stage. **m** Lactyation and H3K18la levels were detected in normal and tumor tissues by Western blot

Since ocular melanoma shows active glycolysis (Additional file 1: Figure S1A-B), which can lead to the production of large amounts of lactate as substrates for histone lactylation, we examined the protein lactylation levels in ocular melanoma and their conceivable clinical significance. Immunofluorescence staining of 82 ocular melanoma tissues and 28 normal melanocyte tissues showed significantly higher levels of global lactylation in ocular melanoma tissues than in normal melanocyte tissues (*p* < 0.05) (Fig. 1a, b, Additional file 2: Table S1). And higher global lactylation levels predicted earlier recurrence and enhanced aggressiveness of cancer (log-rank test, *p* < 0.05) (Fig. 1c, d, Additional file 2: Table S1). Consistently, western blot assays showed that the global lactylation levels were also elevated in most ocular melanoma cell lines (Fig. 1e, f, lanes 2–9). Intriguingly, we noticed that the lactylated proteins were mainly localized in cell nucleus from immunofluorescence staining and the predominant band in western blot assays was near 17 kDa. We then performed silver staining-mass spectrometry (MS) after immunoprecipitation of lactylated proteins, and the results showed that this band was histone H3 (Fig. 1g). Therefore, we decided to investigate whether the above phenomenon was caused by H3K18la. Similarly, H3K18la levels showed the same trend in ocular melanoma tissues compared to normal melanocyte tissues as global lactylation levels did (Fig. 1i, j, Additional file 2: Table S1), and an elevated H3K18la level indicated a poorer prognosis (log-rank test, *p* < 0.05) (Fig. 1k, l, Additional file 2: Table S1). Accordingly, higher H3K18la levels were also observed in most ocular melanoma cell lines (Fig. 1e, lanes 2–9, and h). Both the elevated global lactylation and H3K18la levels in ocular melanoma tissues were confirmed by western blot assays (Fig. 1m, lanes 4–9). These data indicate that a large portion of ocular melanoma is characterized by elevated histone lactylation, which is likely to be involved in the tumorigenesis of ocular melanoma.

### Inhibition of histone lactylation suppressed tumorigenesis of ocular melanoma

**Fig. 2:**
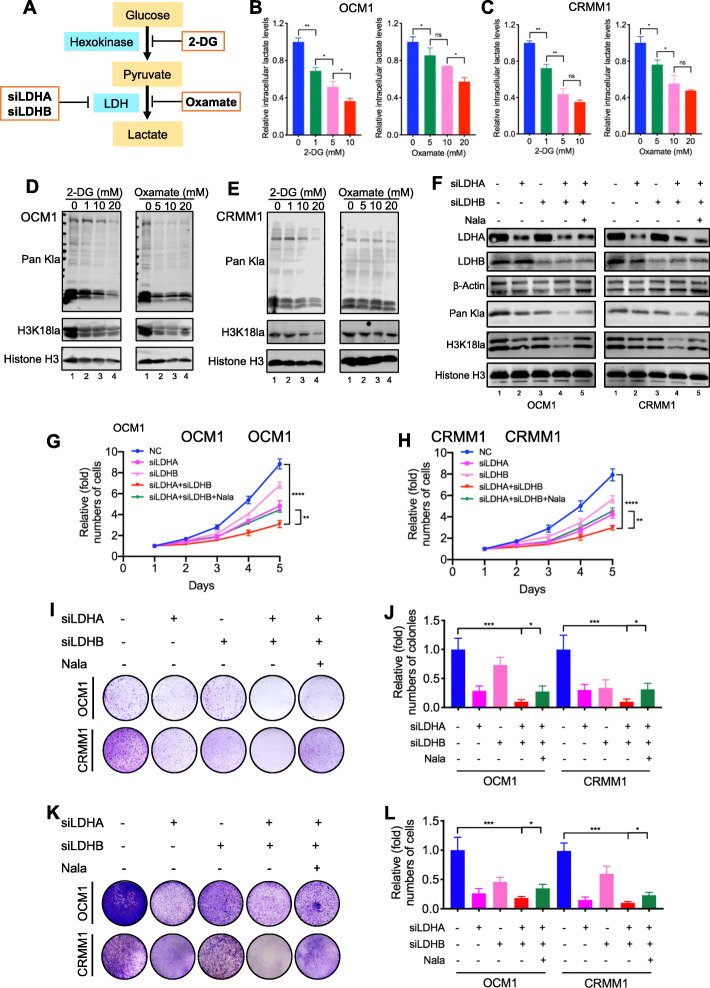
Simultaneous silencing LDHA and LDHB reduced lactyation levels and inhibited ocular melanoma tumorigenesis. **a** Schematic diagram of histone lactylation inhibition methods target. **b, c** Intracellular lactate levels were measured from OCM1 (**b**) and CRMM1 (**c**) cells cultured in different concentrations of 2-DG or oxamate for 24 h by a lactate colorimetric kit. Statistical significance was determined using one-way ANOVA followed by Sidak’s multiple comparisons test, *p < 0.05, **p < 0.01. **d, e** Lactyation and H3K18la levels were detected in OCM1 (**d**) and CRMM1 (**e**) cells cultured in different concentrations of 2-DG or oxamate for 24 h by Western blot. **f** Lactyation and H3K18la levels were detected in OCM1 and CRMM1 cells after LDHA and LDHB silencing by Western blot. **g**, **h** Proliferation of OCM1 (**g**) and CRMM1 (**h**) cells after LDHA and LDHB silencing was analyzed using CCK8 assay. **p < 0.01, ****p < 0.0001. **i** Tumor growth of OCM1 and CRMM1 cells after LDHA and LDHB silencing was evaluated by colony formation assay. **j** Statistical analysis of the colony formation assay performed using OCM1 and CRMM1 cells after LDHA and LDHB silencing. All of the experiments were performed in triplicate, and relative colony numbers are shown as means ± SD. *p < 0.05, ***p < 0.001. **k** The migratory ability of OCM1 and CRMM1 cells after LDHA and LDHB silencing was evaluated by transwell assay. **l** Statistical analysis of cells in the transwell assay performed using OCM1 and CRMM1 cells after LDHA and LDHB silencing. All of the experiments were performed in triplicate, and relative cell numbers are shown as means ± SD. *p < 0.05, ***p < 0.001

To evaluate whether histone lactylation inhibition attenuated tumorigenesis in ocular melanoma, we reduced global intracellular histone lactylation level in tumor cells. Herein, (1) glycolysis inhibitors [the non-metabolizable glucose analog 2-deoxy-d-glucose (2-DG) and oxamate], as well as (2) siRNAs for lactate dehydrogenase (LDHA and LDHB), were adopted to attenuate lactate production and histone lactylation as reported [11] (Fig. 2a). Notably, both glycolysis inhibitors achieved a significant dose-dependent decrease in intracellular lactate (Fig. 2b, c), as well as global lactylation and H3K18la levels (Fig. 2d, e) in ocular melanoma cells (OCM1 and CRMM1 cells). Next, we found that decreased histone lactylation efficiently inhibited ocular melanoma cells proliferation (Additional file 1: Figure S2A). Notably, oxamate presented a higher killing threshold in control cells, PIG1 (Additional file 1: Figure S2A), indicating a tumor-specific killing effect. Moreover, the colony assay demonstrated that the ocular melanoma cells with lower histone lactylation levels developed fewer and smaller colonies (Additional file 1: Figure S2B-E). The transwell and wound-healing assays showed that the migration ability of these cells was weaker (Additional file 1: Figure S3A-D, Figure S3E-H). In vivo, we established orthotopic xenografts in nude mice using glycolysis inhibitor-pretreated OCM1 cells. As expected, the tumor weight in the reduced histone lactylation group was significantly lower than that in control group (Additional file 1: Figure S4A-B). Since 2-DG and oxamate may harbor other effects independent of inhibiting lactate production and lactylation, we further silenced LDHA and LDHB to inhibit histone lactylation. Notably, we found either LDHA-deficient (Fig. 2f, lane 2) or LDHB-deficient (Fig. 2f, lane 3) ocular melanoma cells did not present with remarkable reduction of global histone lactylation. However, simultaneous silencing LDHA and LDHB (Fig. 2f, lane 4) significantly impaired histone lactylation and, in parallel, triggered a significant inhibition of cellular proliferation (Fig. 2g–j) and migration (Fig. 2k, l). In addition, adding back sodium lactate (Nala) into LDHA/LDHB-deficient cells successfully increased histone lactylation levels (Fig. 2f, lane 5) and partially restored cellular proliferation (Fig. 2g–j) and migration (Fig. 2k, l).

Next, sodium lactate, glucose, and rotenone [11] were used to elevate histone lactylations in ocular melanoma cells (OCM1 and CRMM1 cells) (Additional file 1: Figure S5A-B). However, we did not observe significant changes in cell growth (Additional file 1: Figure S5C-D) or colony formation ability (Additional file 1: Figure S5E-H) of ocular melanoma cells after upregulating histone lactylation levels. Taken together, upregulation of histone lactylation could not further promote tumor progression in ocular melanoma cells. This may be due to elevated levels of histone lactylation in the ocular melanoma reaching saturation, which had been sufficient for tumorigenesis. In parallel, in histone lactylation overexpressed normal melanocytes (Additional file 1: Figure S6A), we did not observe significant changes in cell growth (Additional file 1: Figure S6B) or colony formation ability (Additional file 1: Figure S6C-D). These data indicated simply that upregulating histone lactylation could not transform normal cells into tumors. Although elevated histone lactylation was required for tumor progression of ocular melanoma, multi-factor cooperation is warranted during tumorigenesis.

### Identification of potential downstream targets of histone lactylation

**Fig. 3:**
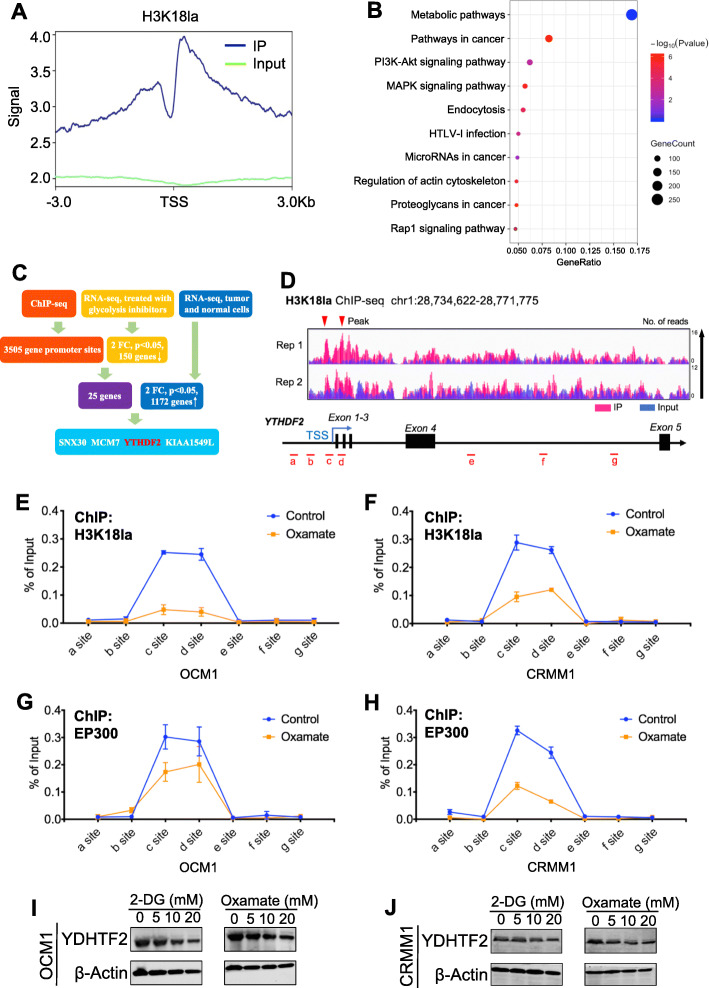
Histone lactyation activated transcription of YTHDF2. **a** Distribution of H3K18 la sites relative to translation start site (TSS). **b** Kyoto Encyclopedia of Genes and Genomes (KEGG) analysis of H3K18 la peaks. **c** Bioinformatics analysis filtered YTHDF2 as a downstream target of H3K18la. **d** IGV tracks for YTHDF2 from ChIP-seq analysis. Sites **a**–**g** are distributed in the YTHDF2 genomic region, and sites **c** and **d** are the H3K18la peaks. **e, f** ChIP-qPCR assay of H3K18la status in the YTHDF2 genomic region in OCM1 (**e**) an CRMM1 (**f**) cells. **g, h** ChIP-qPCR assay of EP300 status in the YTHDF2 genomic region in OCM1 (**g**) and CRMM1 (**h**) cells. **i, j** Western blot were performed to test YTHDF2 expression in OCM1 (**i**) and CRMM1 (**j**) cells after treated with different concentrations of 2-DG or oxamate

To uncover the regulatory role of histone lactylation in gene expression, we first performed chromatin immunoprecipitation followed by sequencing (ChIP-seq) using anti-H3K18la antibodies. Our ChIP-seq data showed that H3K18la was enriched in promoter regions (Fig. 3a, Additional file 1: Figure S7A). A Kyoto Encyclopedia of Genes and Genomes (KEGG) analysis revealed that these H3K18la-specific genes were enriched in metabolic pathways and tumor-related pathways (Fig. 3b), suggesting a regulatory effect of histone lactylation in tumorigenesis. KEGG analysis of downregulated genes after oxamate treatment was also enriched in the metabolism-related pathway (Additional file 1: Figure S7B). Next, by combining ChIP-seq data (GEO accession number: GSE156674) with RNA sequencing (RNA-seq) data of the transcriptome of cells treated with glycolysis inhibitors or not (GEO accession number: GSE156675) and the transcriptome of normal and tumor cells (GEO accession number: GSE137675), we identified 4 anti-H3K18la-ChIP target genes with significantly reduced mRNA levels in cells treated with glycolysis inhibitors and also highly expressed in tumor cells (Fig. 3c). Among these candidate genes, YTHDF2, one of m^6^A readers, has been reported to act as an oncogene in several tumors [16, 17]; therefore, we decided to focus on it. To confirm that transcription of YTHDF2 is activated by H3K18la, we first observed a significant enrichment of the H3K18la signal at the YTHDF2 promoter (Fig. 3d). ChIP-qPCR assays also indicated that H3K18la was enriched in YTHDF2 promoter regions and that this enrichment was reduced by glycolysis inhibitors (Fig. 3e, f). In addition, ChIP-qPCR assays showed that the EP300, a histone lactylation writer [11], binding level was significantly decreased at the YTHDF2 promoter after treatment with glycolysis inhibitors (Fig. 3g, h). However, global protein expression of EP300 remained unchanged in YTHDF2-deficent cells (Additional file 1: Figure S7C). Notably, H3K27ac level was slightly decreased at the YTHDF2 promoter under this condition (Additional file 1: Figure S7D-E). As expected, both mRNA (Additional file 1: Figure S7F-G) and protein (Fig. 3i, j) level of YTHDF2 expression was significantly decreased after treatment with glycolysis inhibitors. Furthermore, the mRNA stability assays demonstrated that glycolysis inhibitors did not affect mRNA stability of YTHDF2 (Additional file 1: Figure S7H-I). In addition, we noticed YTHDF2 expression remained unaltered after simultaneously silencing EP300 and glycolysis inhibition, compared with EP300-deficent or glycolysis inhibition group (Additional file 1: Figure S7J). Notably, we found that YTHDF2 expression was positively correlated with global histone lactylation levels (*R* = 0.475, *P* < 0.001) and H3K18la levels (*R* = 0.393, P < 0.001) in ocular melanoma cohort (Additional file 1: Figure S8A-B). Collectively, these data suggested that the transcription of YTHDF2 is positively regulated by H3K18la.

### YTHDF2 acts as a novel oncogene in ocular melanoma

**Fig. 4:**
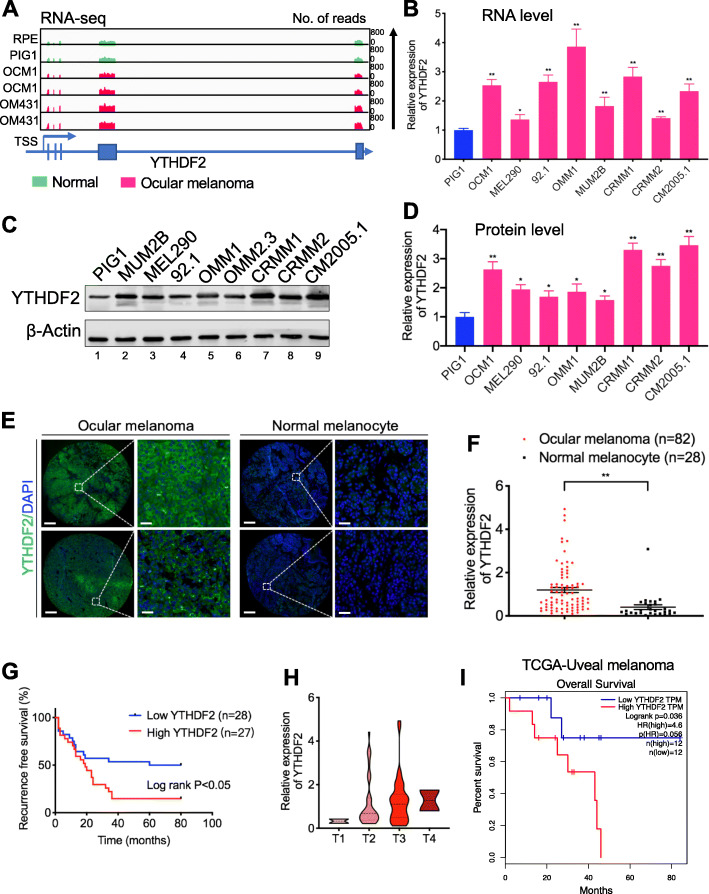
Aberrant YTHDF2 expression in ocular melanoma correlated with prognosis. **a** IGV tracks for YTHDF2 expression from RNA-sequence analysis normal cell lines (PIG1 and RPE) and ocular melanoma cell lines (OCM1 and OM431). **b** Expression of YTHDF2 was detected in normal melanocytes and ocular melanoma cell lines by RT-PCR. *p < 0.05, **p < 0.01. **c** Expression of YTHDF2 was detected in normal melanocytes and ocular melanoma cell lines by Western blot. **d** Densitometric analysis was performed to quantify and statistically compare YTHDF2 levels in normal melanocytes and ocular melanoma cell lines that normalized to β-Actin. *p < 0.05, **p < 0.01. **e, f** YTHDF2 expression in normal and tumor tissues. **e** YTHDF2 expression was visualized by immunofluorescence staining in normal and tumor tissues. Scale bar: left panel, 100 μm; right panel, 20 μm. **b** Statistical results of YTHDF2 expression in normal and tumor tissues. Unpaired *t* test, **p < 0.01. **g** Kaplan-Meier curves of time for recurrence showing the difference between ocular melanoma patients with low and high YTHDF2 expression. “High YTHDF2” was defined as relative YTHDF2 expression level of the samples (determined by luminance intensity) higher than 0.72, while those lower than 0.72 as “low YTHDF2.” *n* = 55, log-rank test, *p* < 0.05. **h** YTHDF2 expression in patients at AJCC stages T1 to T4. There were 2 ocular melanoma samples in T1 stage, 36 in T2 stage, 42 in T3 stage, and 2 in T4 stage. **i** The TCGA database of uveal melanoma demonstrated prolonged survival time in patients with low YTHDF2 expression

**Fig. 5:**
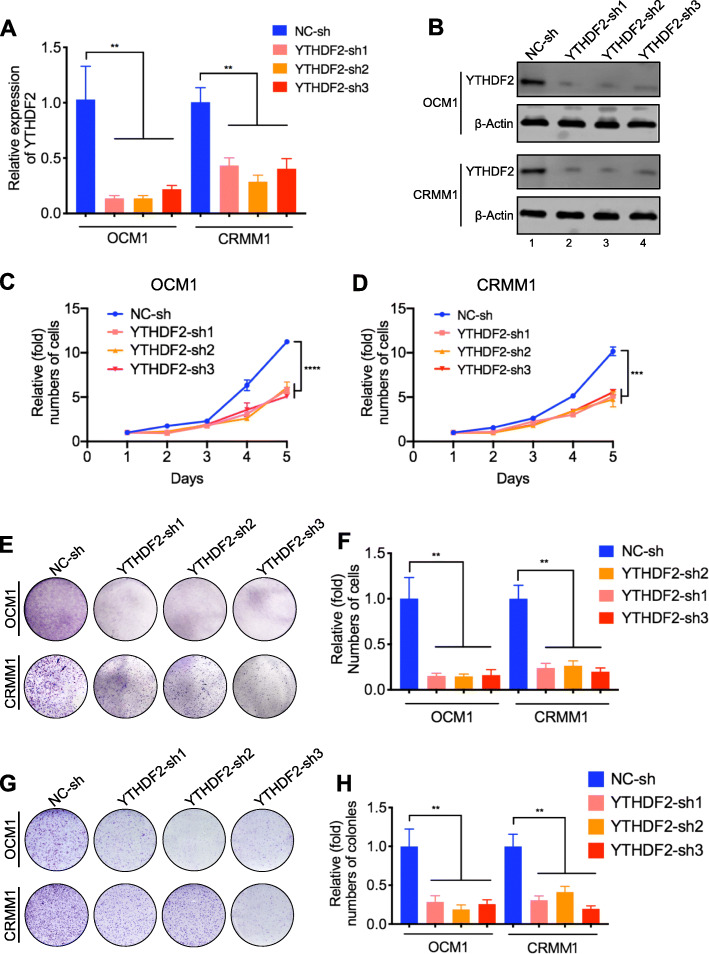
YTHDF2 acted as an oncogene in ocular melanoma cells. **a, b** RT-PCR (**a**) and Western blot (**b**) showed that YTHDF2 was silenced after shRNAs transfection in ocular melanoma cells. **p < 0.01. **c**, **d** Proliferation of OCM1 (**c**) and CRMM1 (**d**) cells with or without YTHDF2 knockdown was analyzed by CCK8 assay. ***p < 0.001, ****p < 0.0001. **e** Tumor growth of OCM1 and CRMM1 cells with or without YTHDF2 knockdown was evaluated by colony formation assay. **f** Statistical analysis of the colony formation assay performed using OCM1 and CRMM1 cells with or without YTHDF2 knockdown. All of the experiments were performed in triplicate, and relative colony numbers are shown as means ± SD. **p < 0.01. **g** The migratory ability of OCM1 and CRMM1 cells with or without YTHDF2 knockdown was evaluated by transwell assay. **h** Statistical analysis of cells in the transwell assay performed using OCM1 and CRMM1 cells with or without YTHDF2 knockdown. All of the experiments were performed in triplicate, and relative cell numbers are shown as means ± SD. **p < 0.01

Since YTHDF2 can be directly regulated by H3K18la, we next explored its function in ocular melanoma. First, we found that YTHDF2 was highly expressed in ocular melanoma cell lines at both the RNA (Fig. 4a, b) and protein levels (Fig. 4c, d, lanes 2–9). In addition, immunofluorescence staining showed that YTHDF2 expression was significantly upregulated in ocular melanoma tissues compared to normal melanocyte tissues (*p* < 0.05) (Fig. 4e, f, Additional file 2: Table S1) and that higher expression of YTHDF2 was positively correlated with a poor prognosis (log-rank test, *p* < 0.05) (Fig. 4g, h, Additional file 2: Table S1). The Gene Expression Profiling Interactive Analysis (GEPIA) database confirmed that a high YTHDF2 level was associated with a poorer prognosis (Fig. 4i). We then verified the function of YTHDF2 in ocular melanoma cells by silencing its expression with three short hairpin RNAs (referred as shYTHDF2-1, shYTHDF2-2 and shYTHDF2-3). After YTHDF2 was successfully knocked down (Fig. 5a, b), we performed CCK-8 assays, which showed that cell growth was remarkably reduced compared to that of control cells (Fig. 5c, d). In addition, we observed that YTHDF2 knockdown inhibited tumor cell colony formation and migration, as measured by plate colony formation assays and transwell assay, respectively (Fig. 5e, h). Taken together, these data indicated that YTHDF2 acts as an oncogene in ocular melanoma.

### Histone lactylation promotes cancer through the m^6^A reader YTHDF2

**Fig. 6:**
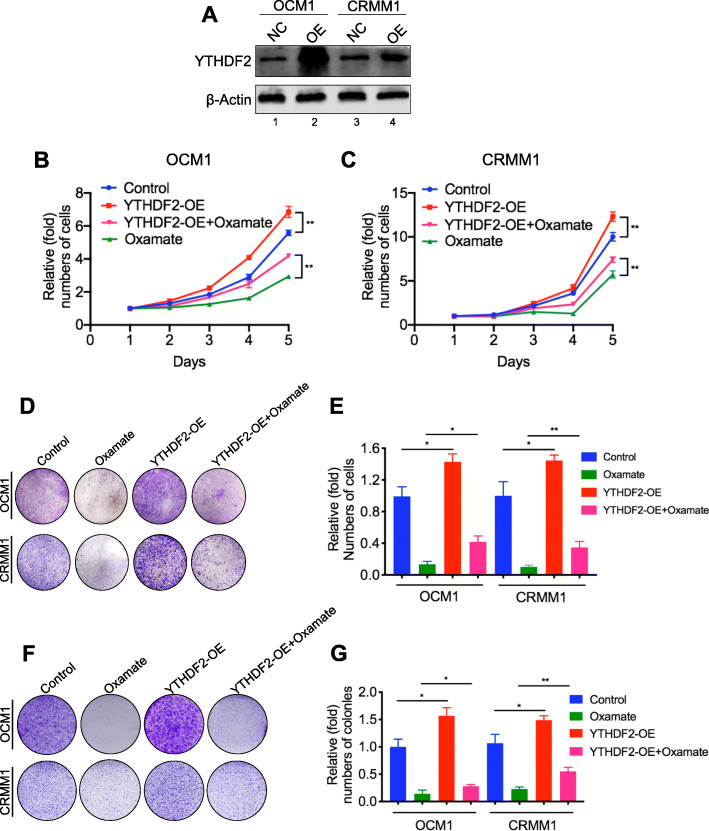
Anticancer effects of glycolysis inhibitors could be partially prevented by YTHDF2. **a** Western blot showed that YTHDF2 was overexpressed in ocular melanoma cells after transfected with plasmid carrying YTHDF2 gene. **b, c** Proliferation of OCM1 (**b**) and CRMM1 (**c**) cells with or without YTHDF2 overexpression treated with oxamate was analyzed by CCK8 assay. **p < 0.01. **d** Tumor growth of OCM1 and CRMM1 cells with or without YTHDF2 overexpression treated with oxamate was evaluated by colony formation assay. **e** Statistical analysis of the colony formation assay performed using OCM1 and CRMM1 cells with or without YTHDF2 overexpression treated with oxamate. All of the experiments were performed in triplicate, and relative colony numbers are shown as means ± SD. *p < 0.05, **p < 0.01. **f** The migratory ability of OCM1 and CRMM1 cells with or without YTHDF2 overexpression treated with oxamate was evaluated by transwell assay. **g** Statistical analysis of cells in the transwell assay performed using OCM1 and CRMM1 cells with or without YTHDF2 overexpression treated with oxamate. All of the experiments were performed in triplicate, and relative cell numbers are shown as means ± SD. *p < 0.05, **p < 0.01

After identifying the expression of YTHDF2 was regulated by H3K18la, we would like to determine whether histone lactylation induced tumor suppression could be rescued by gain of YTHDF2. After overexpressing YTHDF2 in ocular melanoma cells (Fig. 6a), the anticancer effects of glycolysis inhibitors were partially compromised, including cell growth (Fig. 6b, c), colony formation (Fig. 6d, e) and migration (Fig. 6f, g). In addition, overexpression of YTHDF2 in ocular melanoma cells accelerated tumorigenesis, which further indicates the oncogenic gain of function of YTHDF2. Collectively, these results suggested that H3K18la promotes tumorigenesis partially through YTHDF2.

### PER1 and TP53 may serve as the key candidate genes associated with YTHDF2

**Fig. 7:**
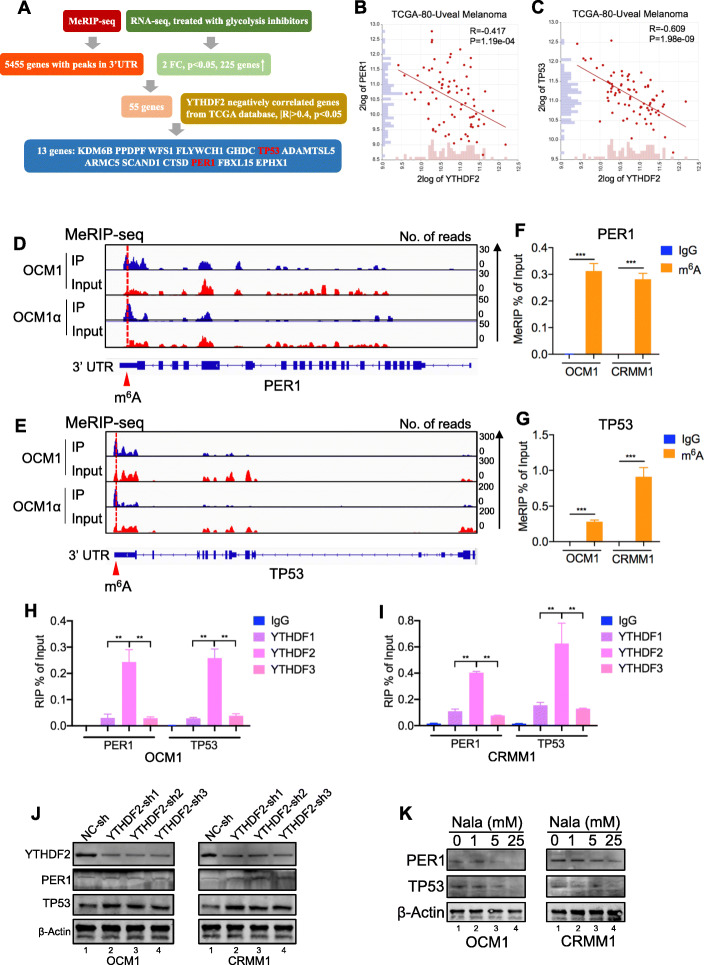
PER1 and TP53 may serve as the key candidate gene of YTHDF2. **a** Bioinformatics analysis filtered PER1 and TP53 may serve as potential downstream target of YTHDF2. **b, c** Gene expression correlation analysis from the TCGA database indicated that YTHDF2 expression negatively correlated with PER1 (*R* = -0.417, *P* = 1.19e−04) (**b**) and TP53 (*R* = − 0.609, *P* = 1.98e−09) (**c**). **d, e** IGV tracks for PER1 (**d**) and TP53 (**e**) from MeRIP-seq analysis in ocular melanoma cells. **f, g** MeRIP-qPCR revealed m^6^A modification of PER1 (**f**) and TP53 (**g**) mRNAs. **h, i** RIP-qPCR analysis revealed that YTHDF2 enriched in PER1 and TP53 transcripts in OCM1 (**h**) and CRMM1 (**i**) cells. **j** Expression of PER1 and TP53 was detected in OCM1 and CRMM1 cells with YTHDF2 knockdown by Western blot. **k** Expression of PER1 and TP53 was detected in OCM1 and CRMM1 cells treated with different concentrations of Nala for 24 h by Western blot

To understand the mechanism of YTHDF2 in tumorigenesis, we explored the R2 (http://hgserver1.amc.nl/cgi-bin/r2/main.cgi) database and found that the genes correlated with YTHDF2 are enriched in tumor-associated biological processes such as DNA repair, apoptosis, and the cell cycle (Additional file 1: Figure S9A). Further KEGG analysis showed that the positively related genes are enriched in pathways such as the RNA degradation pathway and that the negatively related genes are enriched in metabolic pathways (Additional file 1: Figure S9B-C). Next, by combining MeRIP-seq data (GEO accession number: GSE137675) with RNA-seq data of cells treated with glycolysis inhibitors (GEO accession number: GSE156675), we identified that 13 genes with m^6^A peaks in 3′UTR were significantly increased in cells treated with glycolysis inhibitors and negatively correlated (|*R*| > 0.4, *p* < 0.05) with YTHDF2 in the TCGA database (Fig. 7a). Further real-time PCR (RT-PCR) assays showed that 2 of the genes (PER1 and TP53) were remarkably upregulated (foldchange *>* 5, *p <* 0.05) in both two tumor cell lines after treated with glycolysis inhibitors (Additional file 1: Figure S9D-E). Specifically, tumor suppressor genes PER1 [18] (*R* = − 0.417, *P* = 1.19e−04) and TP53 (*R* = − 0.609, *P* = 1.98e−09) were negatively correlated with TYHDF2 in the TCGA database (Fig. 7b, c, Additional file 1: Figure S9F), while oncogenes such as NRAS and BRAF were positively correlated with TYHDF2 (Additional file 1: Figure S9F). We therefore hypothesized that YTHDF2 promotes PER1 and TP53 degradation by binding to their respective m^6^A sites.

**Fig. 8:**
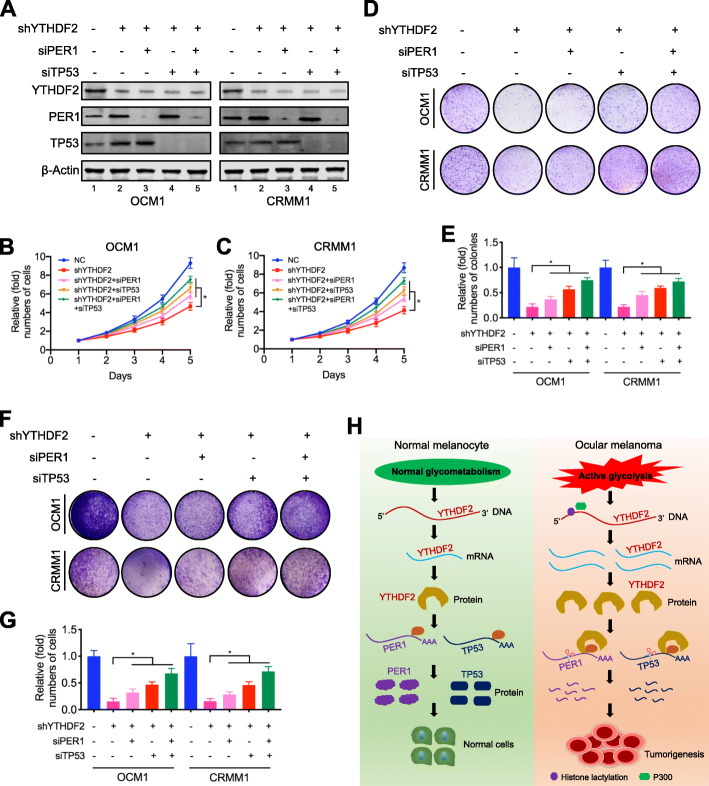
Silencing PER1 and TP53 abolished the YTHDF2 knockdown effect. **a** Western blot showed that PER1 and TP53 was silenced in YTHDF2 knockdown ocular melanoma cells. **b, c** The effect of PER1 and TP53 silencing on proliferation of YTHDF2 knockdown OCM1 (**b**) and CRMM1 (**c**) cells was analyzed using CCK8 assay. *p < 0.05. **d** The effect of PER1 and TP53 silencing on tumor growth of YTHDF2 knockdown OCM1 and CRMM1 cells was evaluated by colony formation assay. **e** Statistical analysis of the colony formation assay performed using YTHDF2 knockdown OCM1 and CRMM1 cells with or without PER1 and TP53 silencing. All of the experiments were performed in triplicate, and relative colony numbers are shown as means ± SD. *p < 0.05. **f** The effect of PER1 and TP53 silencing on the migratory ability of YTHDF2 knockdown OCM1 and CRMM1 cells was evaluated by transwell assay. **g** Statistical analysis of cells in the transwell assay performed using YTHDF2 knockdown OCM1 and CRMM1 cells with or without PER1 and TP53 silencing. All of the experiments were performed in triplicate, and relative cell numbers are shown as means ± SD. *p < 0.05. **h** In ocular melanoma cells, higher histone lactylation level induced by aerobic glycolysis promoted the transcription of YTHDF2, which recognizes the m^6^A modification site on the RNA of two tumor suppressor genes, PER1 and TP53, and promoted their degradation and contributes to the aggressive traits in ocular melanoma progression

MeRIP-seq and meCLIP-seq data (GEO accession number: GSE137675) revealed that PER1 and TP53 had m^6^A sites in the 3′ UTR region (Fig. 7d, e, Additional file 1: Figure S10A-B). MeRIP-qPCR was then applied to prove the m^6^A modification of PER1 and TP53. When compared to the IgG group, an enrichment of PER1 and TP53 mRNA was obtained by the reaction to m^6^A-specific antibody (Fig. 7f, g). As expected, the RIP-qPCR analysis revealed that PER1 and TP53 mRNAs mainly interacted with YTHDF2 (Fig. 7h, i). And YTHDF2 knockdown remarkably upregulated PER1 and TP53 mRNA levels (Additional file 1: Figure S11A-B) and protein levels (Fig. 7j). In addition, we observed PER1 and TP53 were downregulated by histone lactylation inducer (Nala) (Fig. 7k) and upregulated by histone lactylation inhibitors (2-DG and oxamate) (Additional file 1: Figure S11C). We then investigated the PER1 and TP53 mRNA stability alterations in YTHDF2-deficient cells. As a result, YTHDF2 silencing significantly increased mRNA stability of PER1 and TP53 (Additional file 1: Figure S11D-E). Moreover, by exploring the GEPIA database, we observed a higher expression level of PER1 predicted a better prognosis (Additional file 1: Figure S9G). We also observed significantly decreased PER1 and TP53 levels in ocular melanoma tissue samples comparing to control samples (Additional file 1: Figure S12A-C). Next, we examined whether YTHDF2 knockdown effect could be compromised by silencing PER1 and TP53 (Fig. 8a). As a result, silencing PER1 and TP53 partially restored cellular proliferation (Fig. 8b–e) and migration (Fig. 8f, g) in YTHDF2-deficient ocular melanoma cells. Taken together, these data suggest that aberrant YTHDF2-PER1/TP53 axis contributes to tumor progression in ocular melanoma.

## Discussion

To date, cancers have been revealed to present with distinct histone modification patterns, giving rise to an abnormal transcriptome atmosphere in tumorigenesis [19]. Recently, a new type of histone modification, named histone lactylation, was identified [11], but its role in tumors had not been reported. Since ocular melanoma shows active glycolysis that produce large amounts of lactate as substrates for histone lactylation, we explored the underlying role of histone lactylation in tumorigenesis for the first time. We identified that ocular melanoma presented with elevated level of histone lactylation and target correction of aberrant histone lactylation efficiently inhibited tumorigenesis. Elevated histone lactylation promotes the expression of YTHDF2, and YTHDF2 binds to the m^6^A sites of PER1 and TP53 mRNAs for RNA degradation, thereby leading to a novel mechanism for responding to the critical role of histone lactylation in tumorigenesis (Fig. 8h).

Lactate-derived lysine lactylation in histones serves as a new type of histone mark and 28 lactylation sites on core histones have been identified, including H3K4, H3K18, H4K5, and H4K. Increased histone lactylation in promoter regions has been proven to induce the expression of homeostatic genes, including Arg1, during M1 macrophage polarization caused by infection [11]. Here, we for the first time revealed histone lactylation could drive oncogene expression and accelerate tumorigenesis. Since many studies have indicated that the high level of lactase is closely associated with major hallmarks of cancer. Our study should be regarded as an initial exploration and important replenishment of current metabolic-epigenetic understandings. Notably, EP300 is an indicative writer protein for diversified histone acylations, including histone acetylation, lactylation and crotonylation [20]. In our study, we presented a model where EP300 served as a ‘writer’ of lactylation in YTHDF2 promoter, thereby regulating its expression. However, whether other EP300-guided histone modifications participated in YTHDF2 transcriptional regulation requires further explorations.

Dynamic N6-methyladenosine (m^6^A) RNA modification generated and erased by N6-methyltransferases and demethylases regulates gene expression, RNA stability, alternative splicing, and translational efficacy [21]. To date, it has been acknowledged that m^6^A modifications play a key role in the initiation and progression of tumors [22]. For example, aberrant m^6^A modifications of BATF2 mRNA by METTL3 repressed its expression in gastric cancer, which contributes to the tumor growth and metastasis [23]. In hepatocellular carcinoma, HBV-induced m^6^A modifications of PTEN RNA affected innate immunity and contributed to the development of hepatocellular carcinoma [24]. In addition, YTHDF1 promoted non-small cell lung cancer cell proliferation through regulating the translational efficiency of CDK2, CDK4, and cyclin D1 [25]. Here, we for the first time bridged histone lacylation and RNA modifications, which added novel understandings in tumorigenesis.

It should be noted that YTH N6-methyladenosine RNA-binding protein 2 (YTHDF2), an m^6^A reader, was reported to selectively bind to m^6^A sites to regulate mRNA degradation [26]. Accumulating evidence has shown that it is involved in tumorigenesis. It was also proven that YTHDF2 causes m^6^A-mediated SOCS2 mRNA degradation and contributes to liver cancer progression [27]. Moreover, in acute myeloid leukemia, YTHDF2 reduced the half-life of many m^6^A transcripts that promote the overall integrity of leukemic stem cell function, including tumor necrosis factor receptor Tnfrsf2 [17]. In our study, we showed that YTHDF2 contributes to tumorigenesis by degrading PER1 and TP53, and thus YTHDF2 can be considered a novel oncogene in ocular melanoma.

## Conclusions

In summary, our study initially revealed histone lactylation accelerate tumorigenesis through activating m^6^A reader protein, YTHDF2, and thereby provide novel histone lactylation targets for treating ocular melanoma. We also bridged histone modifications with RNA modifications, which provided novel understanding towards epigenetic regulation. Because increased level of lactate is a major hallmark across diversified malignancies, our study provided a novel pattern of metabolic and epigenetic aberration in oncogenesis.

## Methods

### Patients and specimens

We used 88 human ocular melanoma tissues and 28 human normal melanocyte tissues collected from the Department of Ophthalmology, Shanghai Ninth People’s Hospital, Shanghai Jiao Tong University School of Medicine from 2007 to 2017. The clinical and histological features of these specimens have been described previously [28]. The prognostic information was available in 58 tumor samples in our ocular melanoma cohort, while other patients were lost during follow-up. The use of clinical samples with patient consent was approved by the Institutional Research Ethics Committee.

### Cell lines and cell cultures

The 92.1, MUM2B, and OCM1 human ocular melanoma cell lines were kindly provided by Professor John F. Marshall (Tumor Biology Laboratory, Cancer Research UK Clinical Center, John Vane Science Centre, London, UK). The MEL290, OMM1, CRMM1, CRMM2, and CM2005.1 cells were kindly supplied by Professor Martine J. Jager (Department of Ophthalmology, Leiden University Medical Center, Leiden, The Netherlands). The human normal melanocyte cell line PIG1 was a kind gift from the Department of Ophthalmology, Peking University Third Hospital. The HEK293T cell line was purchased from the American Type Culture Collection (Manassas, VA, USA). All cell lines used in our study were authenticated by STR profiling. For culturing, the MUM2B, OCM1, and HEK293T cells were grown in DMEM (Gibco). The 92.1, MEL290, and OMM1 cells were cultured in RPMI 1640 medium (Gibco). CRMM1, CRMM2, CM2005.1, and PIG1 cells were cultured in Ham’s F-12 K (Kaighn’s). The cells are cultured in medium supplemented with 10% certified heat-inactivated fetal bovine serum (FBS; Gibco), penicillin (100 U/mL), and streptomycin (100 mg/mL) at 37 °C in a humidified 5% CO_2_ atmosphere.

### Immunofluorescence

Human ocular melanoma tissues and normal melanocyte-bearing tissues in paraffin-embedded sections were deparaffinized, rehydrated, fixed, and blocked with 5% normal goat serum (Vector) and then incubated with pan anti-Kla (PTM-1401), anti-H3K18la (PTM-1406), and anti-YTHDF2 (24744-1-AP, Proteintech) antibodies at 4 °C overnight. Thereafter, the slides were incubated with the appropriate secondary antibodies for 30 min, and nuclei were counterstained with DAPI (Sigma-Aldrich) for 1 h. Digital images were obtained with a ZEISS Axio Scope A1 upright microscope. Relative pan-Kla levels, H3K18la levels, and YTHDF2 expression were determined by comparing the fluorescence intensity of the target antibody with that of DAPI [target fluorescence intensity (488 nm absorbance)/DAPI intensity (405 nm absorbance)]. The associations between pan-Kla levels, H3K18la levels, YTHDF2 expression, and clinicopathological characteristics are listed in Additional file 3: Table S2.

### Western blotting

Cell and tissue extracts were prepared with RIPA lysis buffer (Biosharp, Hefei, China, BL504A) and centrifuged at 13,000×*g* for 30 min at 4 °C. Then, protein samples were separated by 7.5% (wt/vol) sodium dodecyl sulfate polyacrylamide gel electrophoresis (SDS-PAGE) and transferred to polyvinylidene fluoride membranes (Millipore Corporation, Billerica, MA, USA). After blocking with 5% milk for 1 h at room temperature, the membranes were incubated with primary antibody at 4 °C overnight. The membranes were then incubated with secondary antibodies conjugated to a fluorescent tag (Invitrogen). The band signals were visualized using an Odyssey Infrared Imagining System (LI-COR, USA).

### Cell proliferation assays

Cells (2000–3000) were seeded into 96-well plates (Corning) with 100 μL of complete medium. Cell proliferation was analyzed by CCK-8 assays (Dojido) at 0, 24, 48, and 72 h following the manufacturer’s instructions. Ten microliters of CCK-8 reagent was added to each well at the indicated time points, and after 3–4 h of incubation at 37 °C, the absorbance was detected at a wavelength of 450 nm, and growth curves were generated to determine the growth rates.

### Colony formation assay

A total of 2 mL of complete medium containing 1000 cells was placed in a 6-well plate, and the medium was replaced every 3 to 4 days. The colonies were stained with 0.25% crystal violet after 7–14 days.

### Transwell assay

A 24-well Transwell system (Corning) and polycarbonate filters (8-μm pores, Corning) were used in the Transwell assay. A total of 1.0 × 10^4^–1.5 × 10^5^ cells suspended in medium with 2% FBS were placed in the upper compartment, and 500 μL of complete medium was added to the lower chamber. After 1 day of incubation, the cells in the Transwell system were stained with 0.25% crystal violet. The cells that migrated to the lower chamber were imaged and counted.

### Tumor orthotopic xenografts in nude mouse models

For orthotopic xenograft experiments, we used 4-week-old male BALB/c nude mice in a specific pathogen-free (SPF) animal room. Briefly, nude mice were anesthetized by intraperitoneal injection of a ketamine (final concentration: 10 mg/mL) and xylazine (final concentration: 1 mg/mL) mixture (0.01 mL/g mouse weight). Then, the mouse sclera was pre-perforated using a sharp 30-gauge injection needle. Ocular melanoma cells (5 × 10^5^) were injected through the hole made in the choroid by a 33-gauge blunt-end microinjection needle (7803-05, Hamilton, Reno, NV, USA). Then, the infected eyes were treated with ophthalmic bacitracin ointment. All animal experiments were approved by the Animal Care and Use Committee at Shanghai Jiao Tong University School of Medicine.

### ChIP-seq

ChIP assays were carried out as previously described [29]. One hundred million cells were fixed with 1% formaldehyde and sonicated for 8 min (10 s on and 15 s off) on ice with a 2-mm microtip with a 40% output control and 90% duty cycle setting. To perform ChIP, sonicated chromatin fragments (150 μl) were diluted 10-fold, and Protein G agarose (60 μl) (Millipore, USA) was added and shaken at 4 °C for 2 h. Then, the mixture was briefly centrifuged at 1000 rpm for 5 min at 4 °C, and then, the supernatant was collected into a new tube. Anti-H3K18la (PTM-1406) was added to the supernatant and incubated overnight at 4 °C. Protein A and protein G magnetic beads (60 μl) (Millipore, USA) were retained in the supernatant for 6 h to pull down the protein at 4 °C. The DNA was released from the bound chromatin after cross-linking reversal and proteinase K treatment, precipitated, and diluted in 100 μl of 0.2 M glycine. Purified DNA fragments were constructed and added to ChIP-seq libraries, amplified, and sequenced on an HiSeq 2500 platform (Illumina). The primers used for the qPCR analysis in this study are listed in Additional file 3**: Table S2.**

### RNA extraction, library construction, and Illumina sequencing

Total RNA was extracted from cultured cell samples using TRIzol reagent (Invitrogen, Carlsbad, CA, USA). We confirmed the integrity of the RNA with a 2100 Bioanalyzer (Agilent Technologies, USA) and measured the RNA concentration using a Qubit 2.0 fluorometer with the Qubit RNA assay kit (Life Technologies, Carlsbad, CA, USA). We next prepared libraries from 100 ng of total RNA using an Illumina TruSeq RNA sample prep kit (San Diego, CA, USA), and libraries were sequenced using a Illumina HiSeq 2500 platform (San Diego, CA, USA). The mRNA levels of the unigenes were identified using TopHat v2.0.9 and Cufflinks and normalized by the fragments per kilobase of exon model per million mapped reads (FPKM). We used the criteria of the false discovery rate (FDR) < 0.01 and fold changes < 0.5 or > 2.0 (<− 1 or > 1 log2 ratio value, *P* value < 0.05) to identify differentially expressed genes.

### RNA isolation and quantitative real-time PCR (RT-PCR)

Total RNA was extracted from cultured cells using the EZpress RNA purification kit (B0004) following the manufacturer’s protocol, and complementary DNA (cDNA) was generated using the PrimeScript RT reagent kit (TaKaRa Bio, Otsu, Japan). Quantitative RT-PCR was performed using SYBR Green PCR master mix (Life Technologies) and a RT-PCR system (Applied Biosystems, Irvine, CA, USA). ACTB was used as the control for PCR product quantification and normalization.

### Plasmid construction

We used pLKO.1 and pGMLV vectors in our study. YTHDF2 shRNAs and verified negative control sequences were generated by PCR and then cloned into the pLKO.1 vector. The YTHDF2 overexpression cassette was generated by PCR and cloned into the pGMLV vector and verified by DNA sequencing.

### Lentivirus packaging and generation of stable cell lines

HEK239T cells were transfected with a mixture of 3 μg of plasmid and 3 μg of pMD2.D plasmid and 6 μg of PsPax plasmid using Lipofectamine 2000 (Invitrogen) in Opti-MEM I reduced serum medium (Gibco). Six hours after transfection, the supernatant was replaced with fresh complete medium. The supernatant containing the viruses was collected at 48 and 72 h after transfection, filtered, and then concentrated with Lenti-X Concentrator (TaKaRa). Fresh medium with 25 μl/mL concentrated lentivirus and 10 ng/ml polybrene (Sigma-Aldrich) was added to cells seeded 24 h prior to transfection. Forty-eight hours later, stable cell lines were selected by incubation with 4 mg/mL puromycin (InvivoGen) for 2 weeks and maintained in 1 mg/mL puromycin.

### RNA-binding protein immunoprecipitation (RIP)-qPCR

The RIP experiment was performed using the Magana RIP Quad kit (Millipore) to examine m6A modification or RNA-binding proteins on individual genes according to the manufacturer’s instructions. Briefly, 1.0 × 10^7^ cells were treated with 200 μL of RIP lysis buffer, of which 15 μL of supernatant was used as input, and 150 μL of supernatant was enriched with antibody- or rabbit IgG-conjugated protein A/G magnetic beads in IP buffer supplemented with RNase inhibitors and incubated overnight at 4 °C. After it was washed, the immunoprecipitated RNA was digested, purified, and further analyzed by qPCR.

### TCGA data set

To validate the potential prognostic significance of YTHDF2 and correlate its expression with PER1 and TP53 in ocular melanoma, we queried GEPIA (gepia.cancer-pku.cn) and R2 (http://hgserver1.amc.nl/cgi-bin/r2/main.cgi) to obtain the transcriptional landscape and obtain follow-up information on the ocular melanoma samples.

## Supplementary Information

**Additional file 1: Supplementary Figure 1.** Ocular melanoma cells are active in glycolysis. **Supplementary Figure 2.** Suppressive effect of glycolysis inhibitors on the proliferation of ocular melanoma cells. **Supplementary Figure 3.** Suppressive effect of glycolysis inhibitors on the migration of ocular melanoma cells. **Supplementary Figure 4.** Suppressive effect of glycolysis inhibitors on the proliferation of ocular melanoma in vivo. **Supplementary Figure 5.** Effects of histone lactyation elevation on ocular melanoma cells. **Supplementary Figure 6.** Effects of histone lactyation elevation on control melanocyte. **Supplementary Figure 7.** YTHDF2 is regulated by histone lactylation. **Supplementary Figure 8.** YTHDF2 expression was positively correlated with histone lactylation levels. **Supplementary Figure 9.** Genes correlated with YTHDF2 in uveal melanoma in TCGA database. **Supplementary Figure 10.** miCLIP-seq data of PER1 and TP53 in ocular melanoma cells. **Supplementary Figure 11.** PER1 and TP53 may serve as the key candidate gene of YTHDF2. **Supplementary Figure 12.** PER1 and TP53 expression levels in tissue samples.

**Additional file 2: Table S1.** Clinical characteristics data.

**Additional file 3: Table S2.** Primers used in this study.

**Additional file 4.** Uncropped western blotting analysis.

**Additional file 5.** Review history.

## Data Availability

Raw data reported in this paper, including RNA-seq, ChIP-seq data, have been deposited in the Gene Expression Omnibus database under accession number GSE156674 and GSE156675 [30, 31]. MeRIP-seq, miCLIP-seq, and RNA-seq data have been deposited in the Gene Expression Omnibus database under accession number GSE137675, https://www.ncbi.nlm.nih.gov/geo/query/acc.cgi?acc=GSE137675 [28].
