# Supplementary material for: Histone lactylation drives oncogenesis by facilitating m6A reader protein YTHDF2 expression in ocular melanoma
Source: Genome Biol. 2021 Mar 16;22:85. doi: 10.1186/s13059-021-02308-z (PMC7962360; doi:10.1186/s13059-021-02308-z)
Supplement: Supplementary file 1 — Additional file 1: Supplementary Figure 1. Ocular melanoma cells are active in glycolysis. Supplementary Figure 2. Suppressive effect of glycolysis inhibitors on the proliferation of ocular melanoma cells. Supplementary Figure 3. Suppressive effect of glycolysis inhibitors on the migration of ocular melanoma cells. Supplementary Figure 4. Suppressive effect of glycolysis inhibitors on the proliferation of ocular melanoma in vivo. Supplementary Figure 5. Effects of histone lactyation elevation on ocular melanoma cells. Supplementary Figure 6. Effects of histone lactyation elevation on control melanocyte. Supplementary Figure 7. YTHDF2 is regulated by histone lactylation. Supplementary Figure 8. YTHDF2 expression was positively correlated with histone lactylation levels. Supplementary Figure 9. Genes correlated with YTHDF2 in uveal melanoma in TCGA database. Supplementary Figure 10. miCLIP-seq data of PER1 and TP53 in ocular melanoma cells. Supplementary Figure 11. PER1 and TP53 may serve as the key candidate gene of YTHDF2. Supplementary Figure 12. PER1 and TP53 expression levels in tissue samples. [file 13059_2021_2308_MOESM1_ESM.pdf]

Supplementary Figure 1 Ocular melanoma cells are active in glycolysis

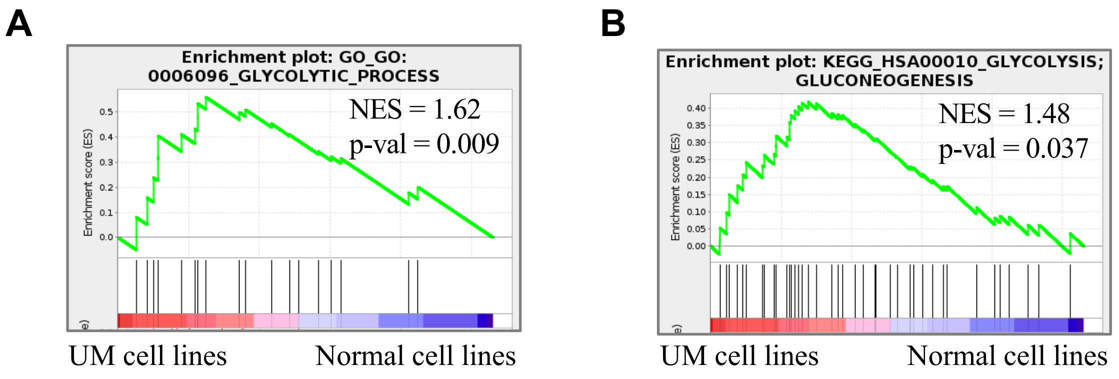

Supplementary Figure 2 Suppressive effect of glycolysis inhibitors on the proliferation of ocular melanoma cells

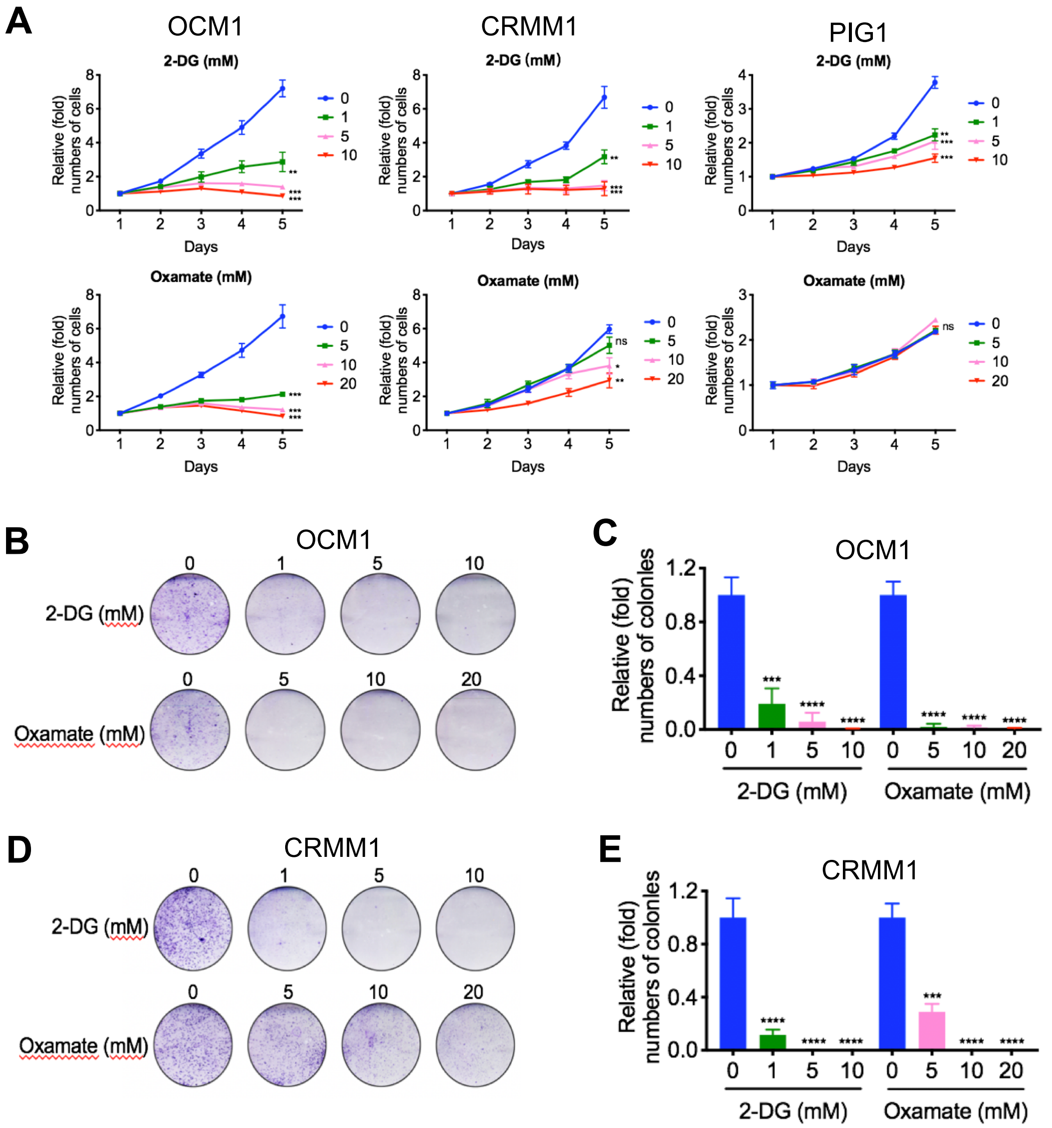

Supplementary Figure 3 Suppressive effect of glycolysis inhibitors on the migration of ocular melanoma cells

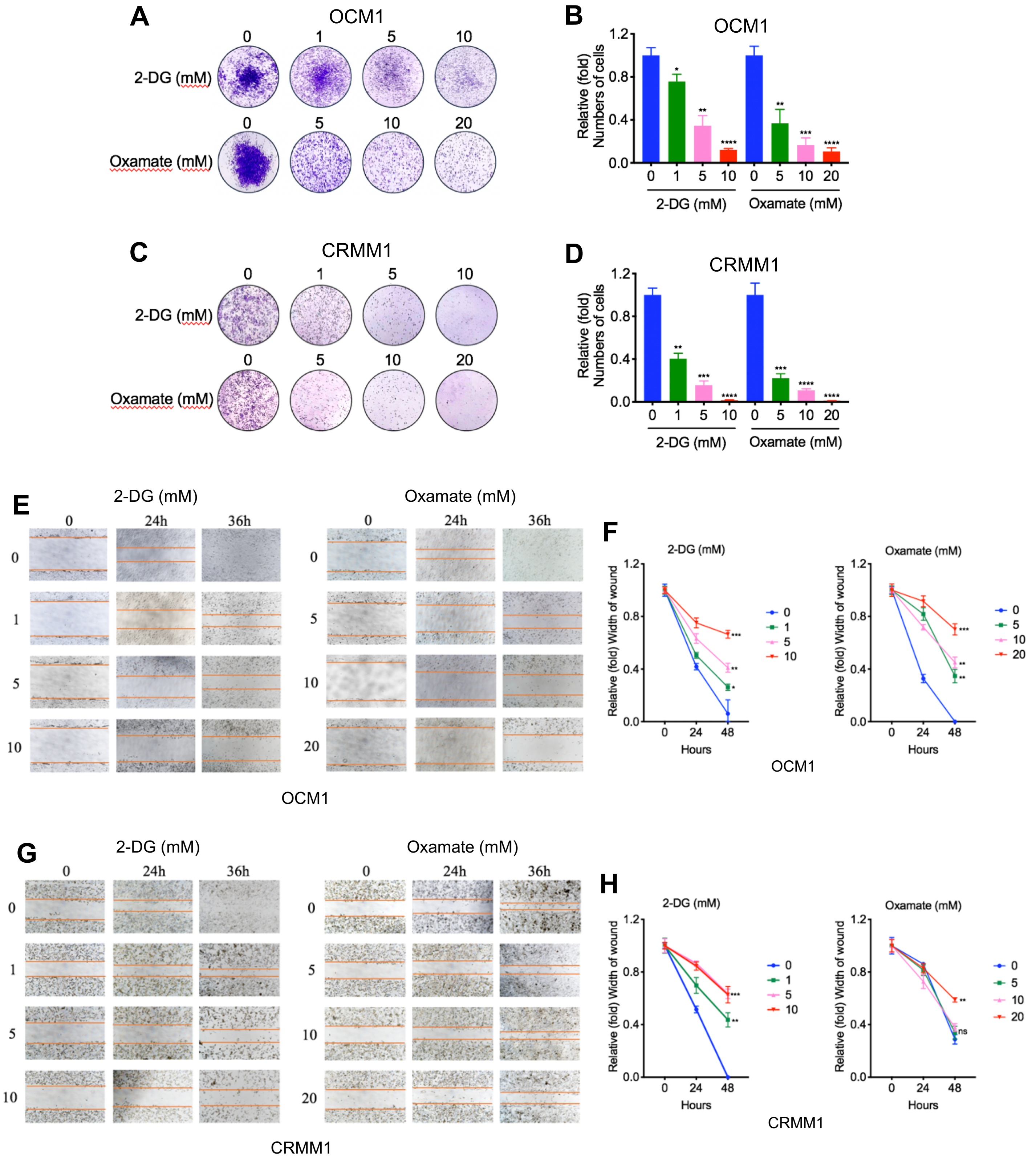

Supplementary Figure 4 Suppressive effect of glycolysis inhibitors on the proliferation of ocular melanoma in vivo

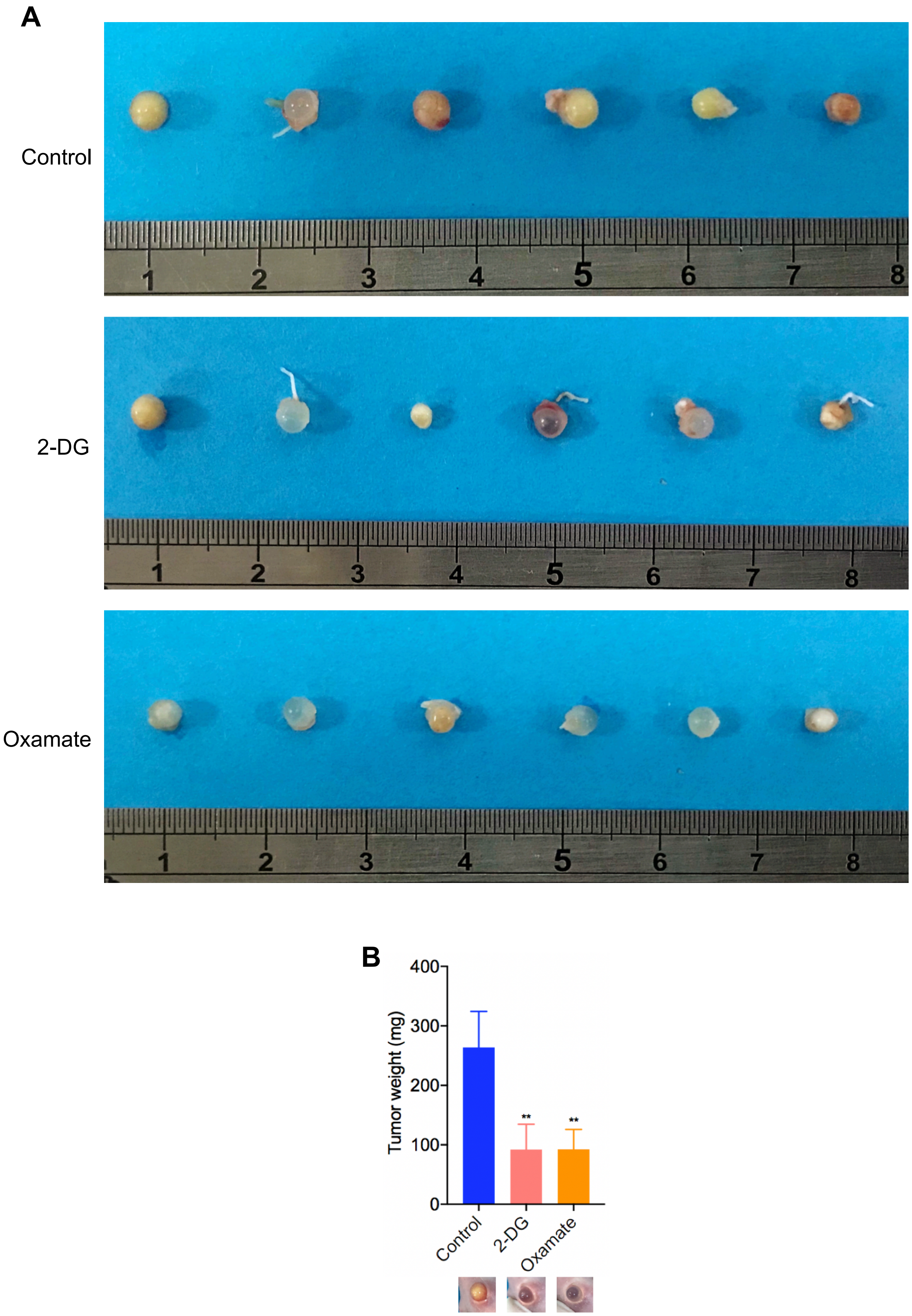

Supplementary Figure 5 Effects of histone lactyation elevation on ocular melanoma cells

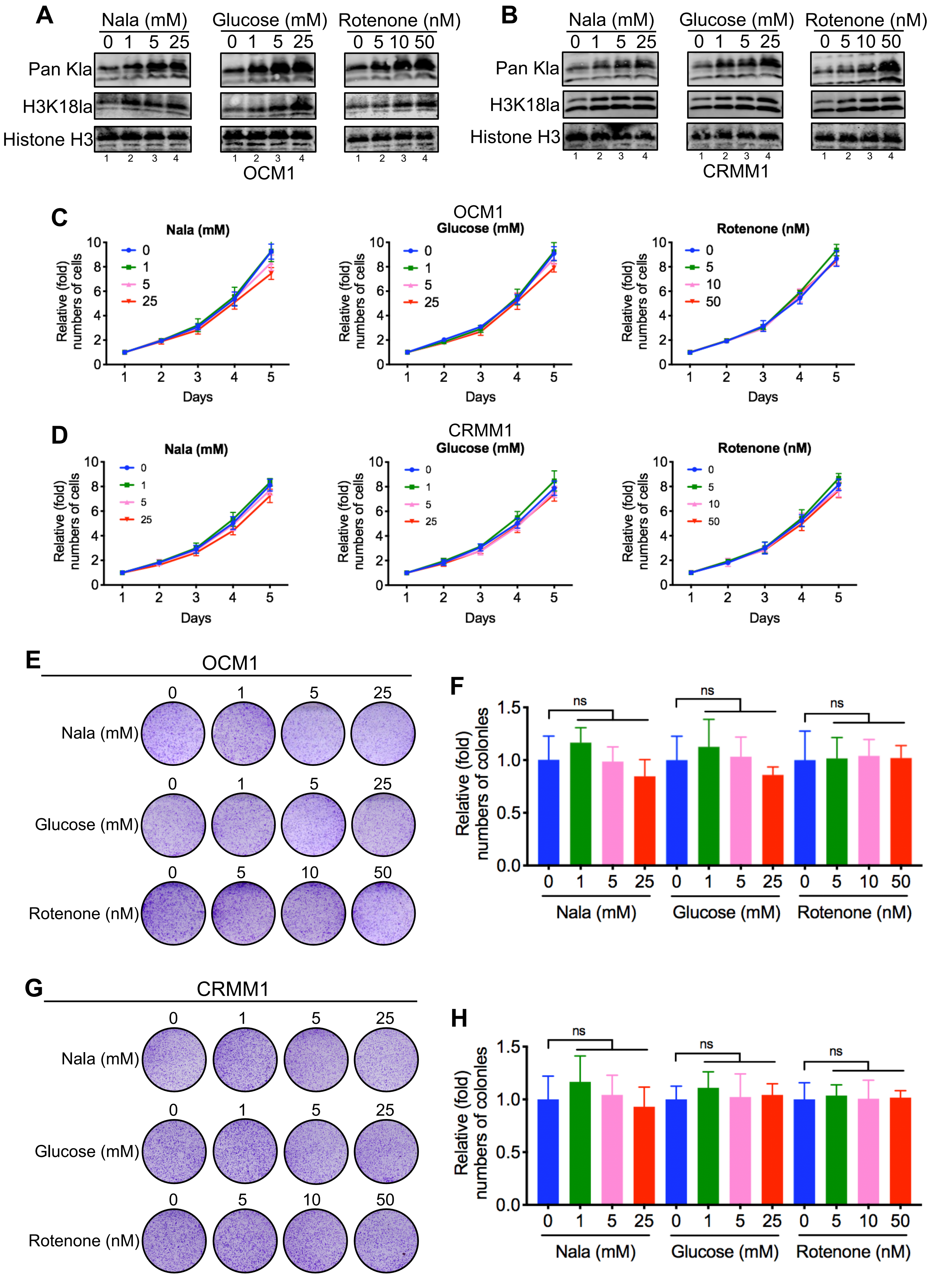

Supplementary Figure 6 Effects of histone lactyation elevation on control melanocyte

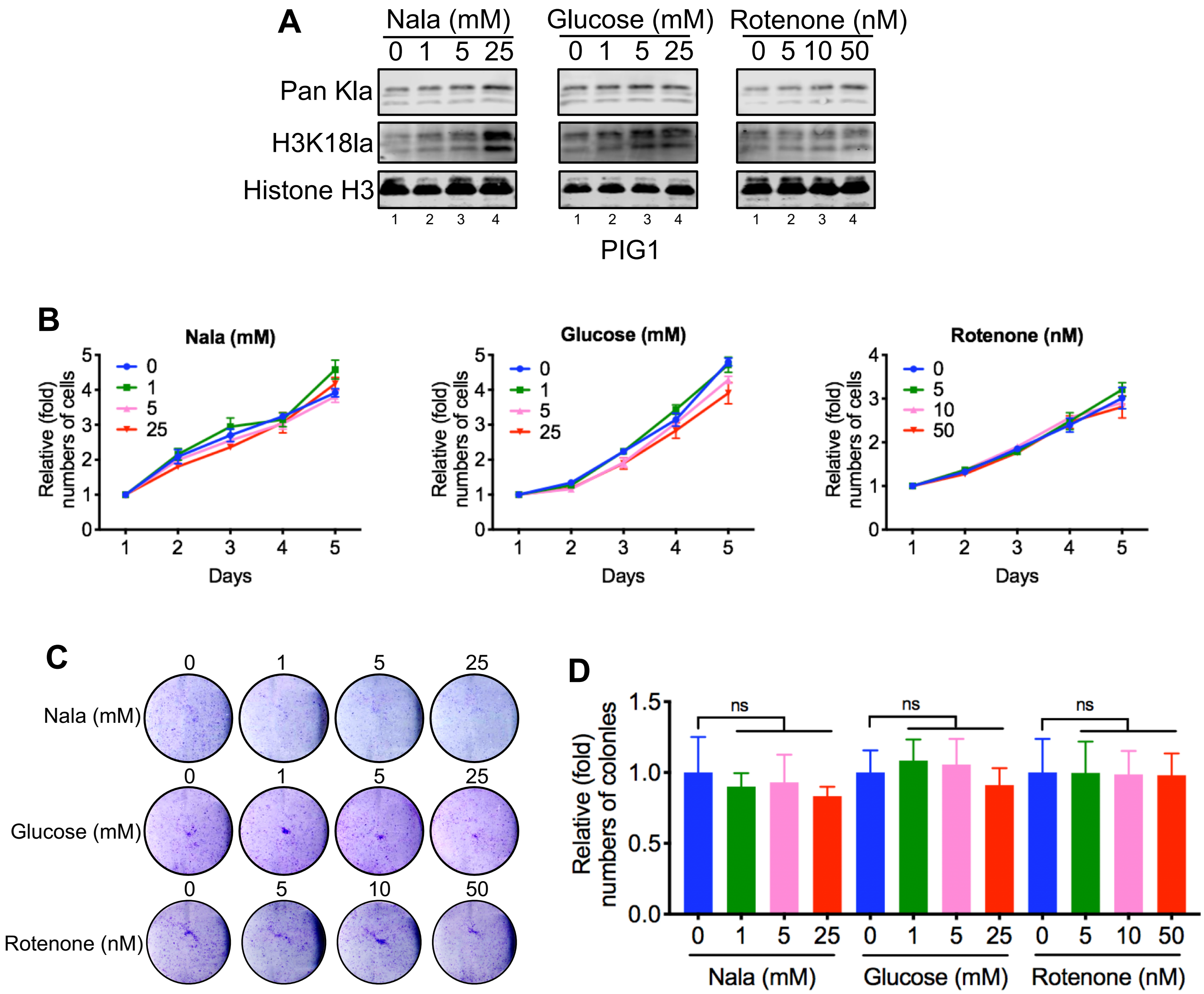

Supplementary Figure 7 YTHDF2 is regulated by histone lactylation

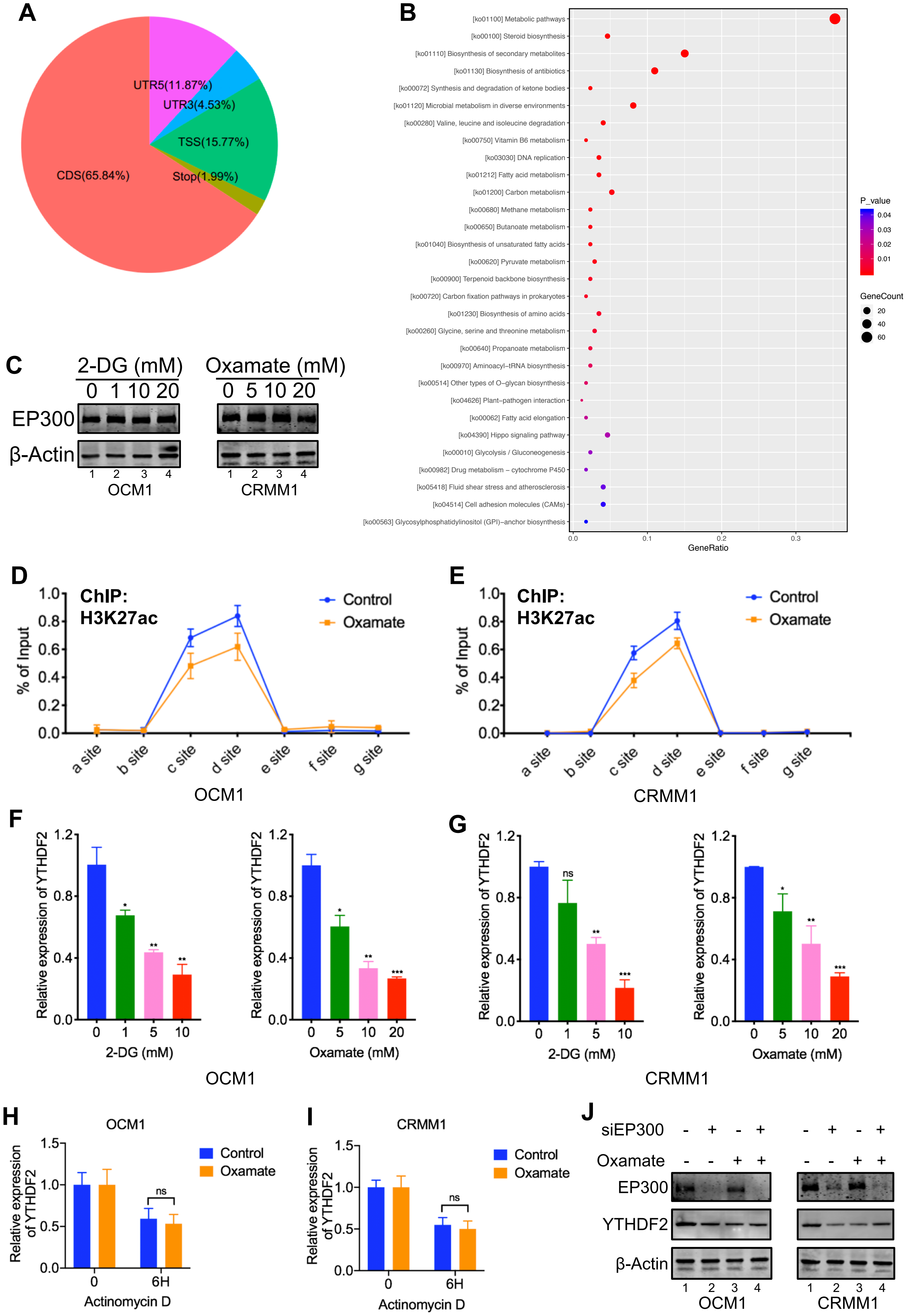

Supplementary Figure 8 YTHDF2 expression was positively correlated with histone lactylation levels

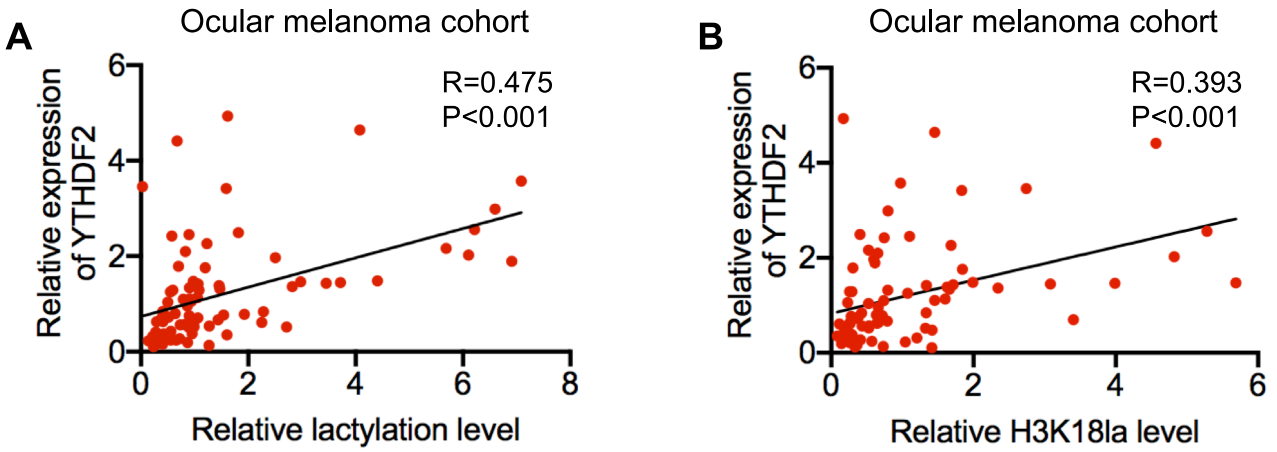

Supplementary Figure 9 Genes correlated with YTHDF2 in uveal melanoma in TCGA database

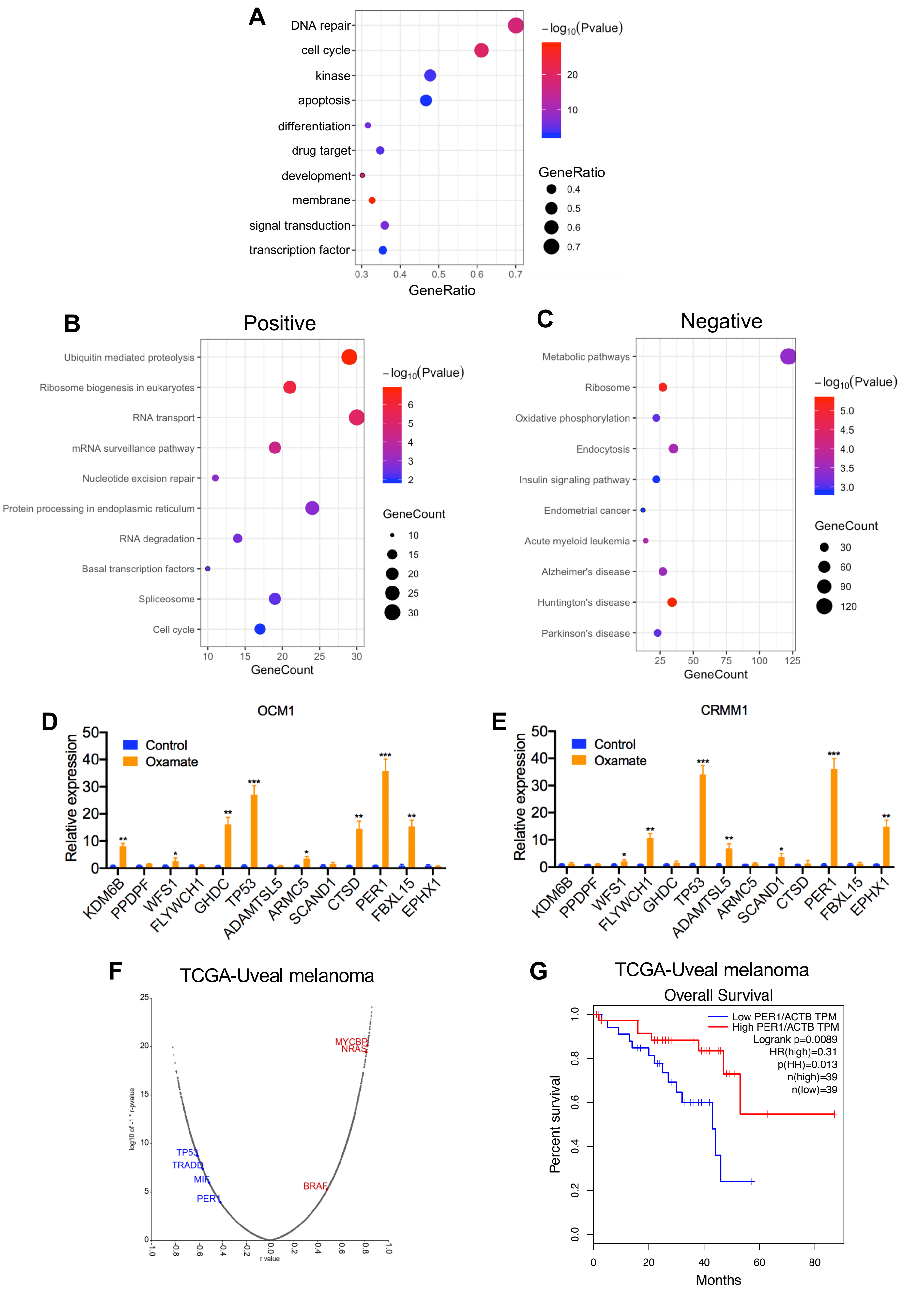

Supplementary Figure 10 miCLIP-seq data of PER1 and TP53 in ocular melanoma cells

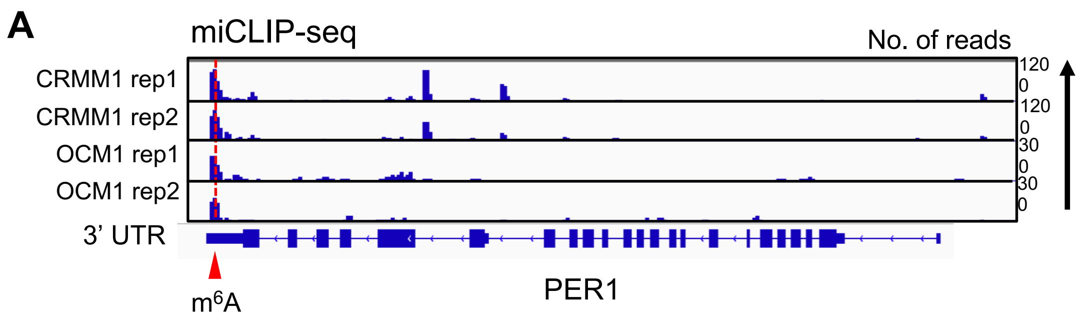

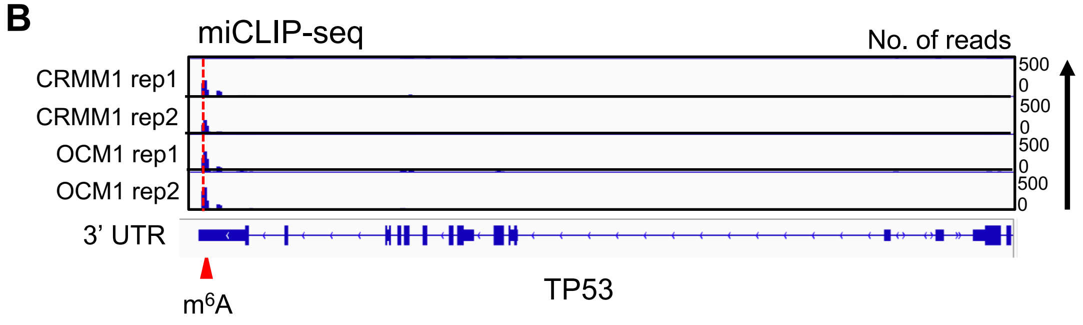

Supplementary Figure 11 PER1 and TP53 may serve as the key candidate gene of YTHDF2

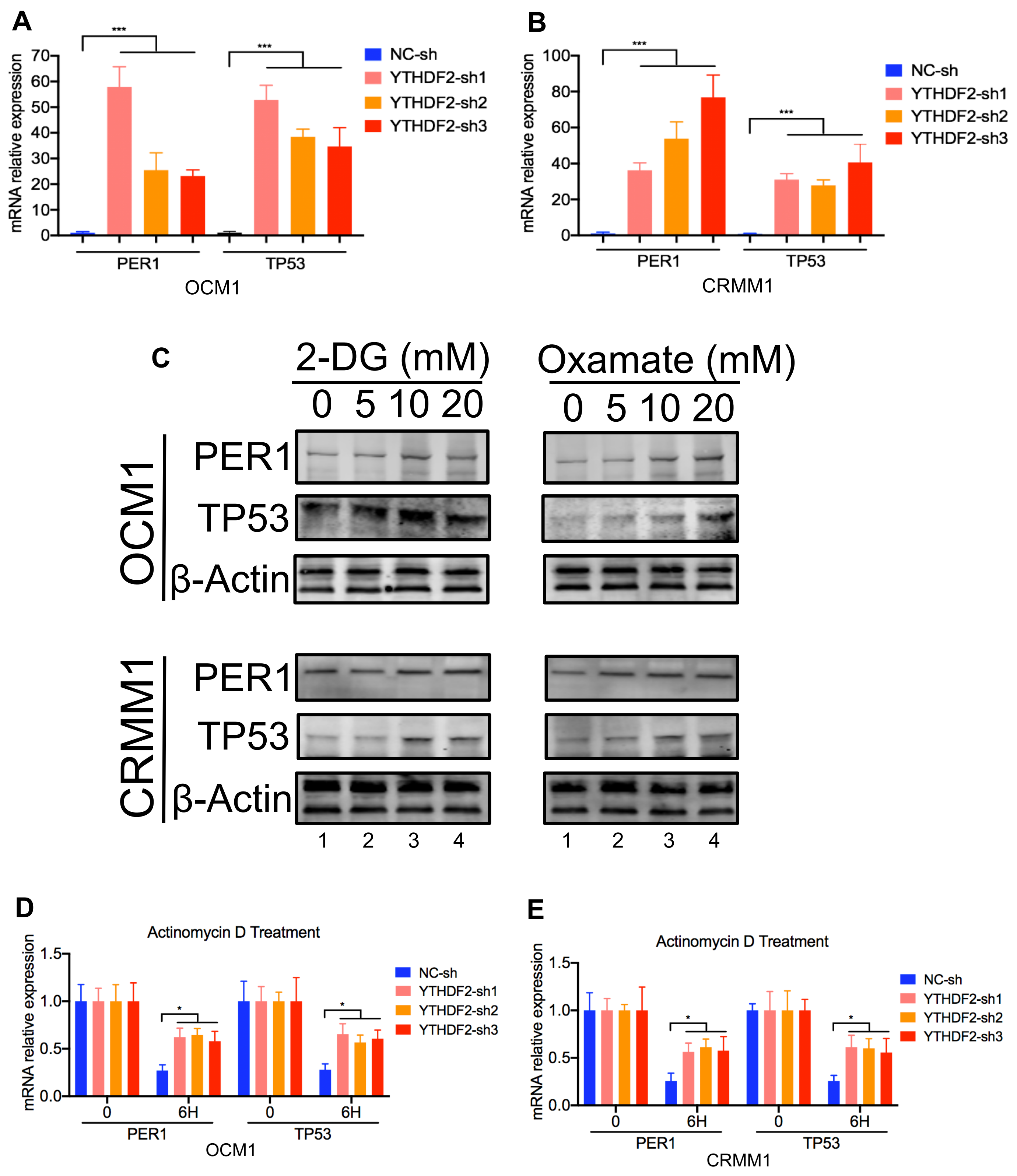

Supplementary Figure 12 PER1 and TP53 expression levels in tissue samples

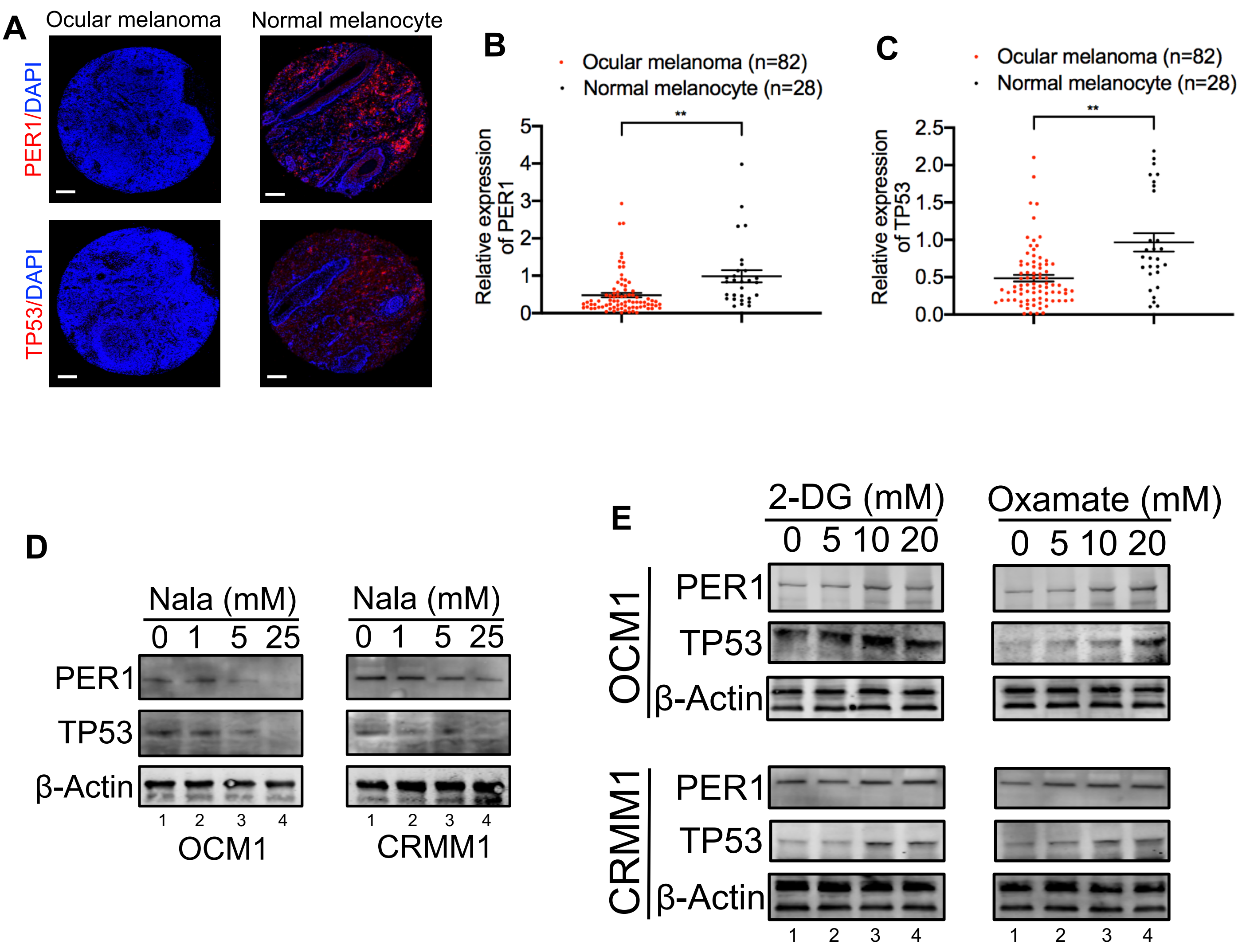

Supplementary Figures

Supplementary Figure 1. Ocular melanoma cells are active in glycolysis

A and B. GSEA plots evaluating the changes in glycolysis in in ocular melanoma cells and normal control cells.

Supplementary Figure 2. Suppressive effect of glycolysis inhibitors on the proliferation of ocular melanoma cells

A. Proliferation of OCM1, CRMM1 and PIG1 cells treated with different concentrations of 2-DG or oxamate was analyzed using CCK8 assay. *p<0.05, **p<0.01, ***p<0.001.

B and D. Tumor growth of OCM1 (B) and CRMM1 (D) cells treated with different concentrations of 2-DG or oxamate was evaluated by colony formation assay.

C and E. Statistical analysis of the colony formation assay performed using OCM1 (C) and CRMM1 (E) cells treated with different concentrations of 2-DG or oxamate. All of the experiments were performed in triplicate, and relative colony numbers are shown as means ± SD. ***p<0.001, ****p<0.0001.

Supplementary Figure 3. Suppressive effect of glycolysis inhibitors on the migration of ocular melanoma cells

A and C. The migratory ability of OCM1 (A) and CRMM1(C) cells treated with different concentrations of 2-DG or oxamate was evaluated by transwell assay.

B and D. Statistical analysis of cells in the transwell assay performed using OCM1 (B)

and CRMM1 (D) cells treated with different concentrations of 2-DG or oxamate. All of the experiments were performed in triplicate, and relative cell numbers are shown as means ± SD. *p<0.05, **p<0.01, ***p<0.001, ****p<0.0001.

E and G. The migratory ability of OCM1 (E) and CRMM1 (G) cells treated with different concentrations of 2-DG or oxamate was evaluated by wound healing assays.

F and H. Statistical analysis of wound healing assays performed using OCM1 (F) and CRMM1 (H) cells treated with different concentrations of 2-DG or oxamate. All of the experiments were performed in triplicate, and relative wound widths are shown as means ± SD. *p<0.05, **p<0.01, ***p<0.001.

Supplementary Figure 4. Suppressive effect of glycolysis inhibitors on the proliferation of ocular melanoma in vivo

A. General pictures of animal demonstrated the suppressive effects of glycolysis inhibitors treated OCM1 cells in orthotopic xenografts.

B. Suppressive effects on tumor weight of glycolysis inhibitors treated orthotopic xenografts. **p<0.01.

Supplementary Figure 5. Effects of histone lactyation elevation on ocular melanoma cells

A and B. Lactyation and H3K18la levels were detected in OCM1 (A) and CRMM1 (B) cells treated with different concentrations of Nala, glucose, or rotenone by Western blot.

C and D. Proliferation of OCM1 (C) and CRMM1 (D) cells treated with different concentrations of Nala, glucose, or rotenone was analyzed using CCK8 assay.

E and G. Tumor growth of OCM1 (E) and CRMM1 (G) cells treated with different concentrations of Nala, glucose, or rotenone was evaluated by colony formation assay.

F and H. Statistical analysis of the colony formation assay performed using OCM1 (F) and CRMM1 (H) cells treated with different concentrations of Nala, glucose, or rotenone. All of the experiments were performed in triplicate, and relative colony numbers are shown as means ± SD.

Supplementary Figure 6. Effects of histone lactyation elevation on control melanocyte

A. Lactyation and H3K18la levels were detected in PIG1 cells treated with different concentrations of Nala, glucose, or rotenone by Western blot.

B. Proliferation of PIG1 cells treated with different concentrations of Nala, glucose, or rotenone was analyzed using CCK8 assay.

C. Tumor growth of PIG1 cells treated with different concentrations of Nala, glucose, or rotenone was evaluated by colony formation assay.

D. Statistical analysis of the colony formation assay performed using PIG1 cells treated with different concentrations of Nala, glucose, or rotenone. All of the experiments were performed in triplicate, and relative colony numbers are shown as means ± SD.

Supplementary Figure 7. YTHDF2 is regulated by histone lactylation

A. The genomic distribution of H3K18la sites.

B. Kyoto Encyclopedia of Genes and Genomes (KEGG) analysis of downregulated genes after oxamate treatment.

C. EP300 levels were detected in OCM1 and CRMM1 cells treated with different concentrations of 2-DG or oxamate by Western blot.

D and E. ChIP-qPCR assay of H3K27ac status in the YTHDF2 genomic region in OCM1 (D) an CRMM1 (E) cells.

F and G. RT-PCR were performed to test YTHDF2 expression in OCM1 (F) and CRMM1 (G) cells after treated with different concentrations of 2-DG or oxamate.

H and I. RT-PCR were performed to test YTHDF2 mRNA stability in OCM1 (H) an CRMM1 (I) cells after treated with oxamate, and Actinomycin D was used to block the transcription of YTHDF2.

Supplementary Figure 8. YTHDF2 expression was positively correlated with histone lactylation levels

A and B. Correlation analysis from ocular melanoma cohort indicated that YTHDF2 expression positively correlated with global histone lactylation levels ($R=0.475$, $P<0.001$) (A) and H3K18la levels ($R=0.393$, $P<0.001$) (B).

Supplementary Figure 9. Genes correlated with YTHDF2 in uveal melanoma in TCGA database

A. Representative GO biological process categories enriched in the cluster of genes correlated with YTHDF2 in uveal melanoma patients in TCGA database.

B and C. Kyoto Encyclopedia of Genes and Genomes (KEGG) analysis of the YTHDF2 expression-positively related genes (B) and the negatively related genes (C) in uveal

melanoma in TCGA database.

D and E. RT-PCR were performed to test potential downstream targets of YTHDF2 obtained by bioinformatics analysis in OCM1 (D) and CRMM1 (E) cells.

F. YTHDF2 correlated genes in uveal melanoma in TCGA database.

G. The TCGA database of uveal melanoma demonstrated prolonged survival time in patients with high PER1 expression.

Supplementary Figure 10. miCLIP-seq data of PER1 and TP53 in ocular melanoma cells

A and B. IGV tracks displaying the miCLIP-seq reads coverage of PER1 and TP53 in OCM1 and CRMM1 cells.

Supplementary Figure 11. PER1 and TP53 may serve as the key candidate gene of YTHDF2

A and B. Expression of YTHDF2 was detected in OCM1 (A) and CRMM1 (B) cells with YTHDF2 knockdown by RT-PCR.

C. Expression of PER1 and TP53 was detected in OCM1 and CRMM1 cells treated with different concentrations of 2-DG and oxamate for 24 h by Western blot.

D and E. RT-PCR were performed to test PER1 and TP53 mRNA stability in OCM1 (D) an CRMM1 (E) cells with YTHDF2 knockdown, and Actinomycin D was used to block the transcription of PER1 and TP53.

Supplementary Figure 12. PER1 and TP53 expression levels in tissue samples

A. PER1 and TP53 expression were visualized by immunofluorescence staining in normal and tumor tissues. Scale bar: 100 μm.

B. Statistical results of PER1 expression in normal and tumor tissues. Unpaired t test, ** $p<0.01$.

C. Statistical results of TP53 expression in normal and tumor tissues. Unpaired t test, ** $p<0.01$.
