# Supplementary material for: Histone lactylation drives oncogenesis by facilitating m6A reader protein YTHDF2 expression in ocular melanoma
Source: Genome Biol. 2021 Mar 16;22:85. doi: 10.1186/s13059-021-02308-z (PMC7962360; doi:10.1186/s13059-021-02308-z)
Supplement: Supplementary file 3 — Additional file 3: Table S2. Primers used in this study. [file 13059_2021_2308_MOESM3_ESM.docx]

**Table S2. Primers used in this study**

**RT-qPCR primers:**

| **Gene** | **Sequences (5’-3’)** |
| --- | --- |
| ACTB-Forward | TGGCACCCAGCACAATGAA |
| ACTB-Reverse | CTAAGTCATAGTCCGCCTAGAAGCA |
| YTHDF2-Forward | TGTTGGAGAAGCTTCGGTCC |
| YTHDF2-Reverse | ACCCGGCCATGTTTCAGATT |
| PER1-Forward | CACTCCTGCGACCAGGTACT |
| PER1-Reverse | TTCTAGGGGGCCACTCATGT |
| TP53-Forward | AAGTCTAGAGCCACCGTCCA |
| TP53-Reverse | CAGTCTGGCTGCCAATCCA |
| KDM6B-Forward | GCGGGTGTTTGTGTTGGAAA |
| KDM6B-Reverse | CGCCTCAGTAACAGCCAGAT |
| PPDPF-Forward | TGGCCACCCACGACTACTA |
| PPDPF-Reverse | CTTCCCGAAAAAGAAGCTGGGG |
| WFS1-Forward | GCCGGATGGACTCCAACACT |
| WFS1-Reverse | TCTCCCTTTGTAGGCCCGGT |
| FLYWCH1-Forward | TGGCTTCACATCCACACAGG |
| FLYWCH1-Reverse | TGTCCAGGCAGCTTTGTCAT |
| GHDC-Forward | AGCAGGGCAAGGAGTATGAG |
| GHDC-Reverse | TATCTGTTCTTCCCCACTCCAGA |
| ADAMTSL5-Forward | GCAACCACCTGGCACTGAT |
| ADAMTSL5-Reverse | AGGAGGACCTGTAGGAGCAG |
| ARMC5-Forward | CCTGACGGTTCAGGCTGAGT |
| ARMC5-Reverse | AGAGAGGGCTTCCGGCAGAT |
| SCAND1-Forward | CAGGGTCAATCAGAAGGGGC |
| SCAND1-Reverse | CAAGTCCCGGACCTTATCCG |
| CTSD-Forward | CTGGACATCGCTTGCTGGAT |
| CTSD-Reverse | AGCACGTTGTTGACGGAGAT |
| FBXL15-Forward | AATGTTACACCACCGGCCAA |
| FBXL15-Reverse | TCCATCGGCTGTCTATTGCG |
| EPHX1-Forward | TCAGAGGGTGAGAACGTGGA |
| EPHX1-Reverse | CTAGCCACATGGCTCCTGTA |

**ChIP-qPCR primers:**

| **Gene** | **Sequences (5’-3’)** |
| --- | --- |
| YTHDF2-site a-Forward | TCGATCTCTCTGGTTTGGTGC |
| YTHDF2-site a-Reverse | AGAGAGAAAAACAAGGCTGGGA |
| YTHDF2-site b-Forward | TTCCCTTCTCTCCTTGCCTG |
| YTHDF2-site b-Reverse | ACAGACAGGTATAACCTCCTCA |
| YTHDF2-site c-Forward | TTATCTTGGGGCCAGCGATT |
| YTHDF2-site c-Reverse | AGAGAGCAAAACACACACCGA |
| YTHDF2-site d-Forward | CCTTTCGGAAGCCGCTTAGT |
| YTHDF2-site d-Reverse | TCCACCCCCACCCTTAATGA |
| YTHDF2-site e-Forward | ACAGGGAGAGTTGGAGCATTTAG |
| YTHDF2-site e-Reverse | CACAGGTTATGCCAAGACACA |
| YTHDF2-site f-Forward | AGCTAGAACGAATGGATAGAGGT |
| YTHDF2-site f-Reverse | GAACCCCCTCTACAACATCCC |
| YTHDF2-site g-Forward | CTCTGAACCATGCCCCTTGTA |
| YTHDF2-site g-Reverse | AACCCTCTGCTTCTAAGGCAA |
