# Supplementary material for: Histone lactylation drives oncogenesis by facilitating m6A reader protein YTHDF2 expression in ocular melanoma
Source: Genome Biol. 2021 Mar 16;22:85. doi: 10.1186/s13059-021-02308-z (PMC7962360; doi:10.1186/s13059-021-02308-z)

Fig1E

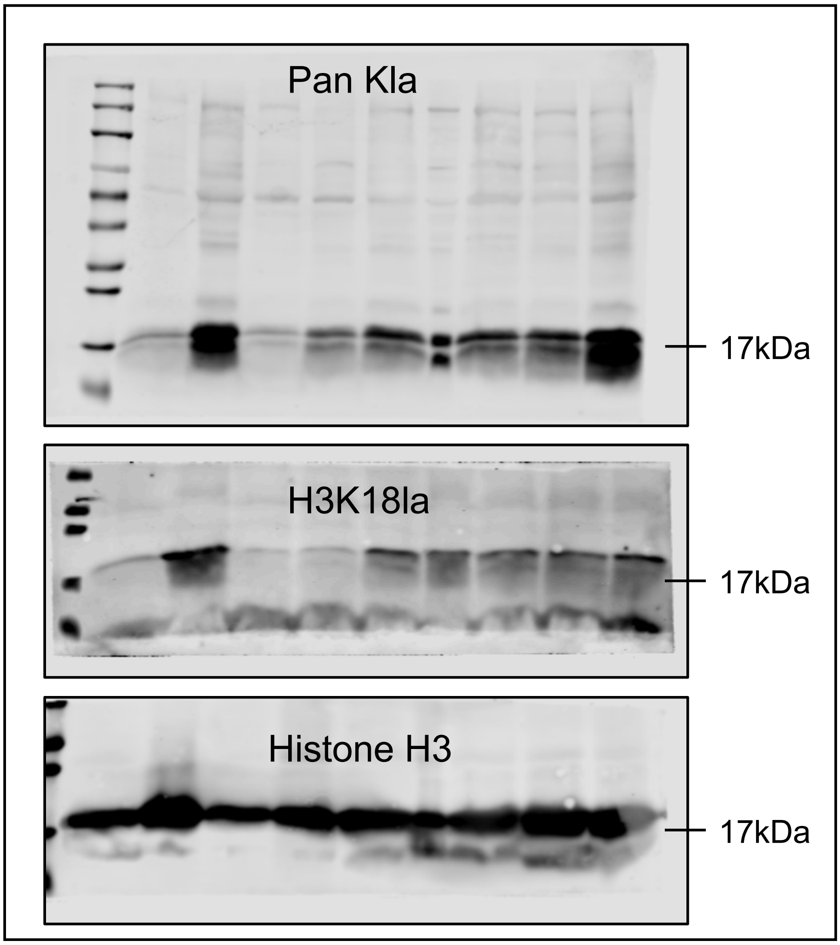

Pan Kla
17kDa
H3K18la
17kDa
Histone H3
17kDa

Fig1M

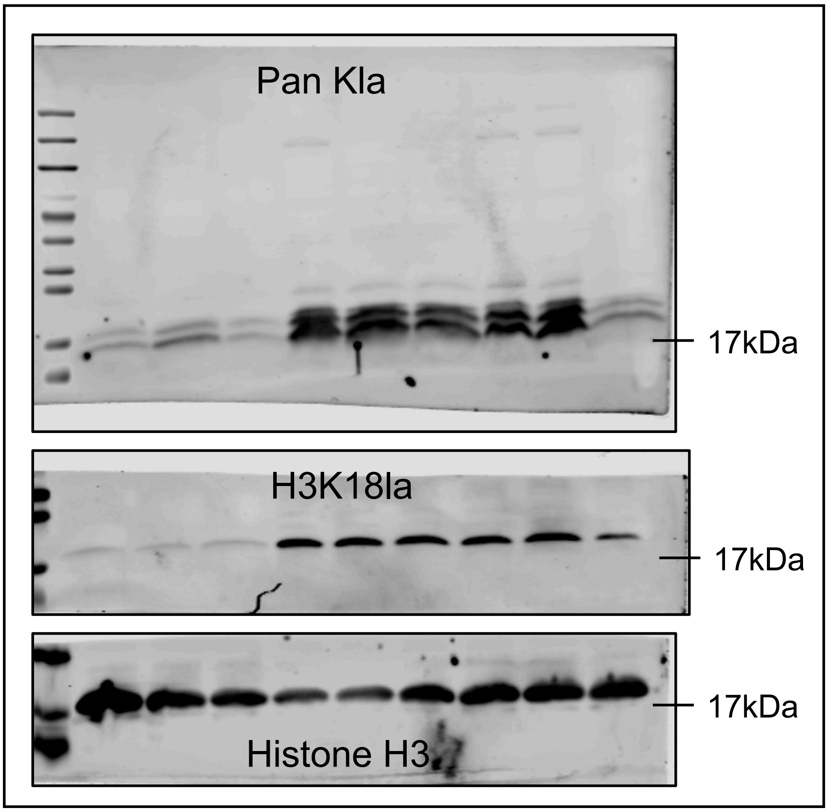

Pan Kla
17kDa
H3K18la
17kDa
Histone H3
17kDa

Fig2D

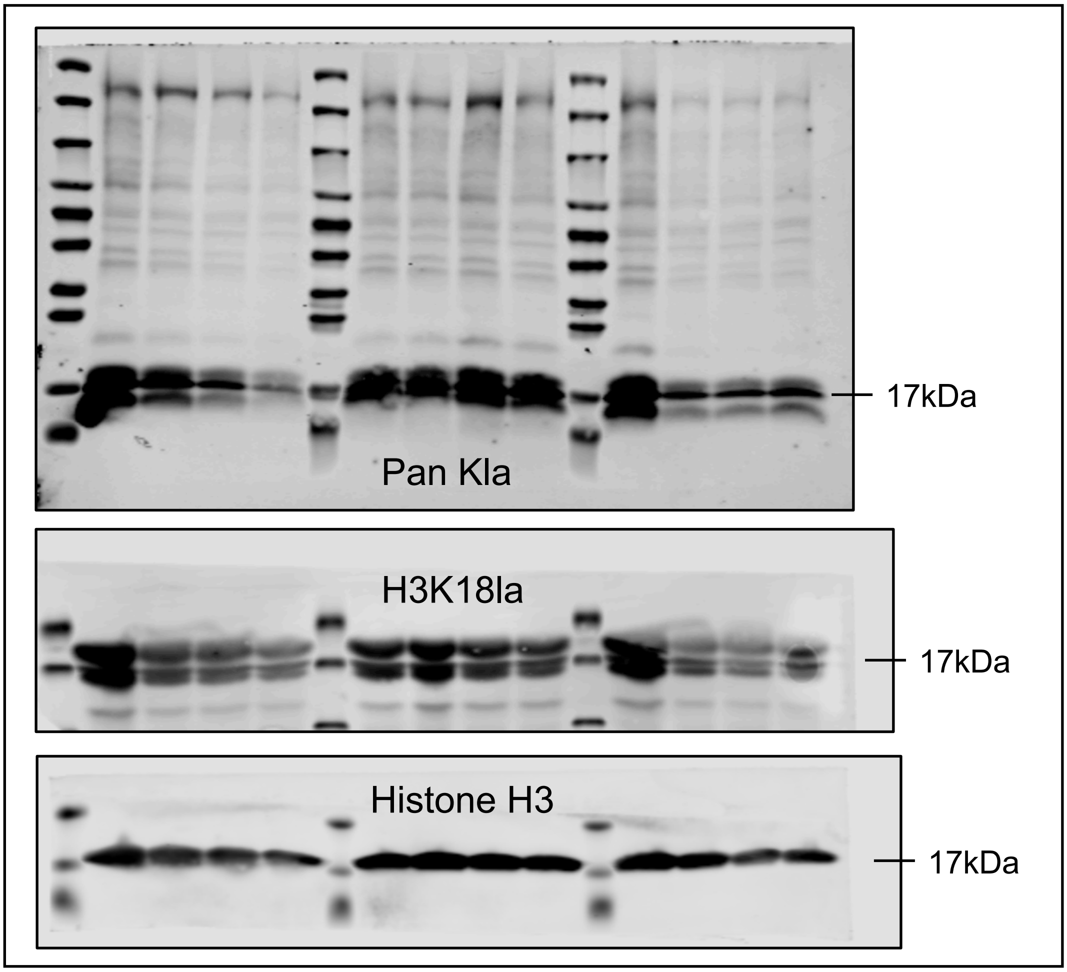

Pan Kla
17kDa
H3K18la
17kDa
Histone H3
17kDa

Fig2E

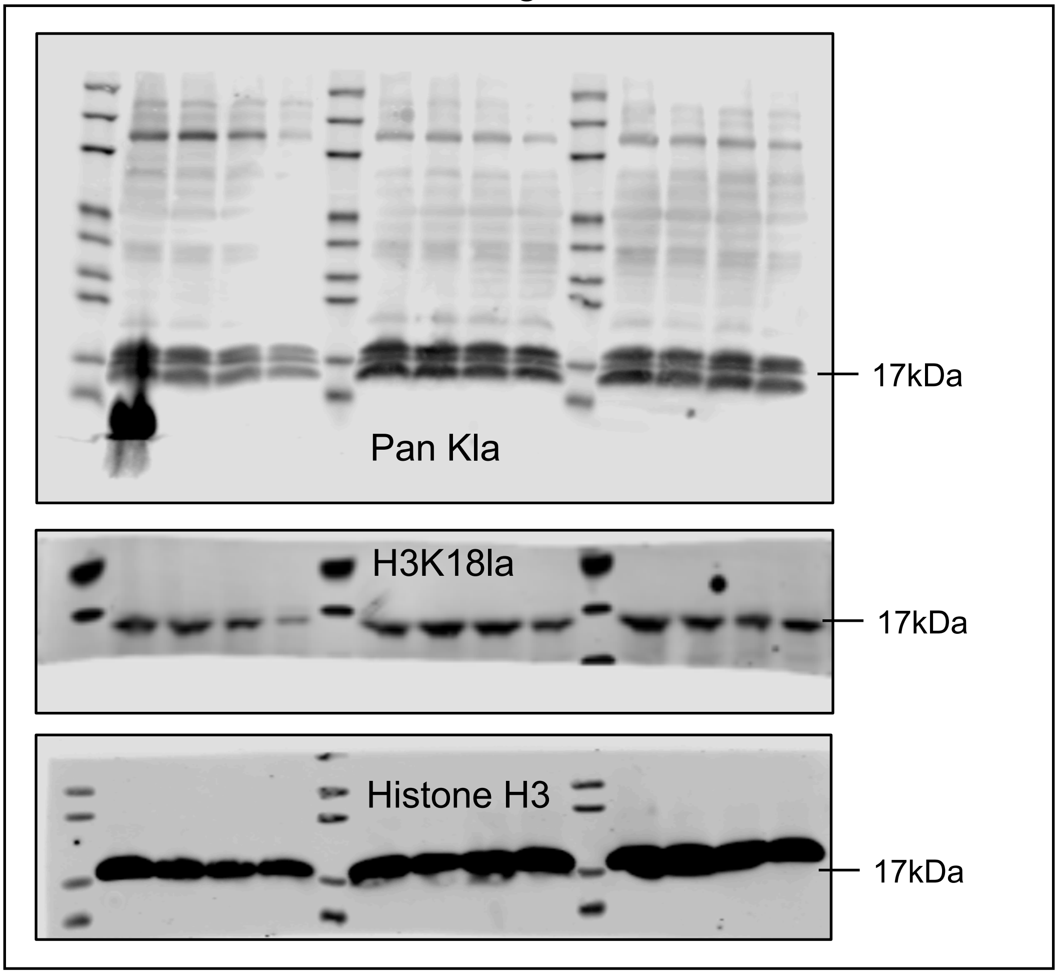

Pan Kla
17kDa
H3K18la
17kDa
Histone H3
17kDa

Fig2F

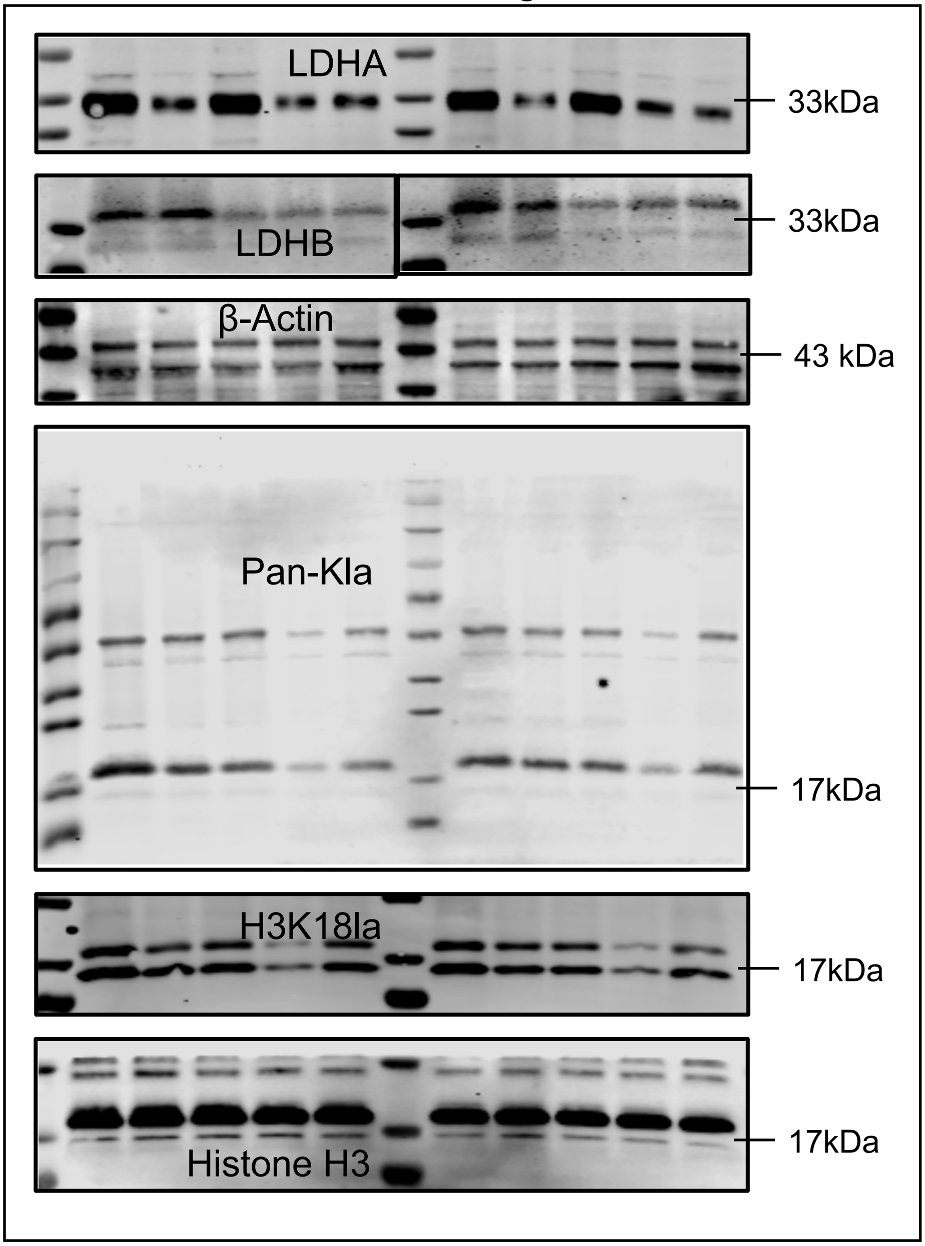
LDHA
33kDa
LDHB
33kDa
β-Actin
43 kDa
Pan-Kla
17kDa
H3K18la
17kDa
Histone H3
17kDa

Fig3I

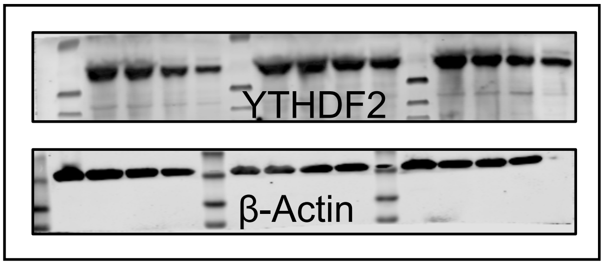

Fig3J

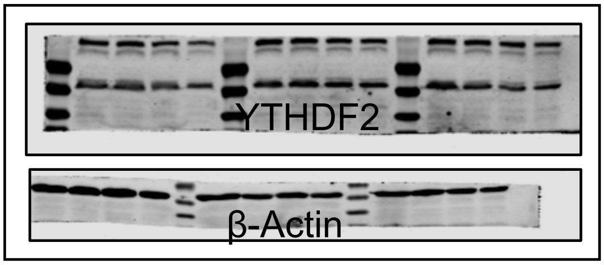

Fig4C

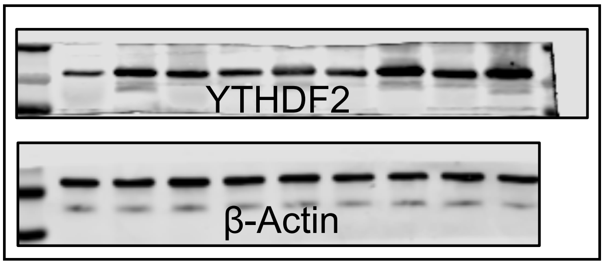

Fig5B

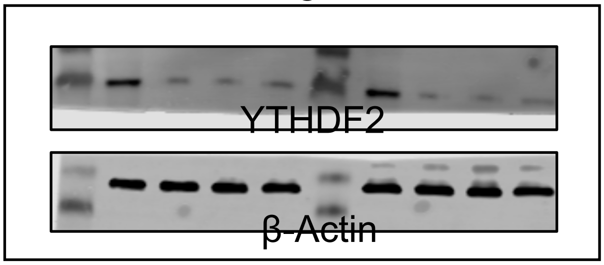

Fig6A

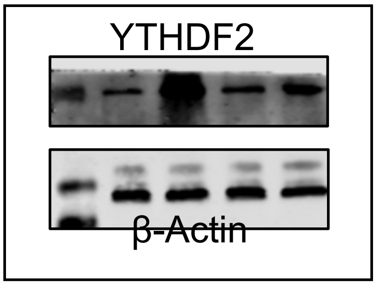

Fig7J

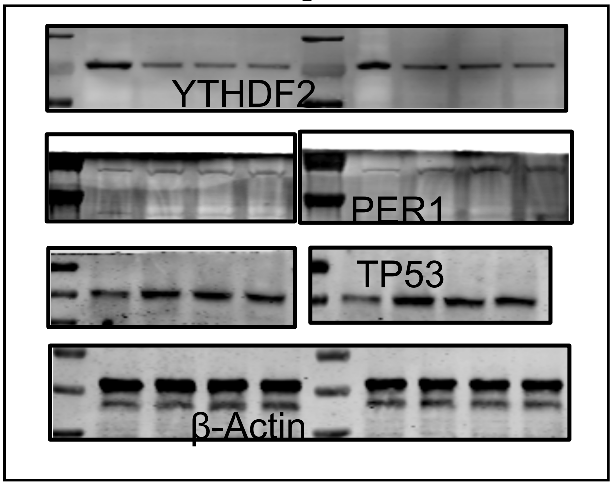

Fig7K

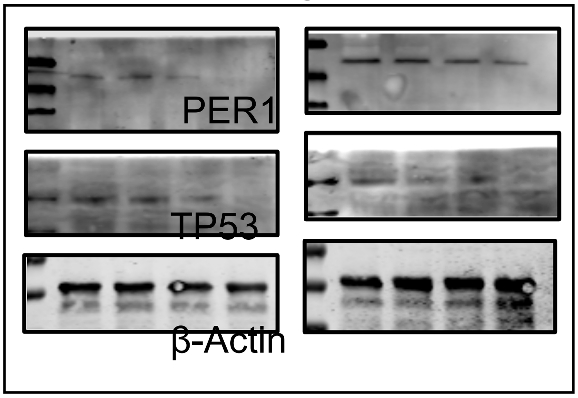

Fig8A

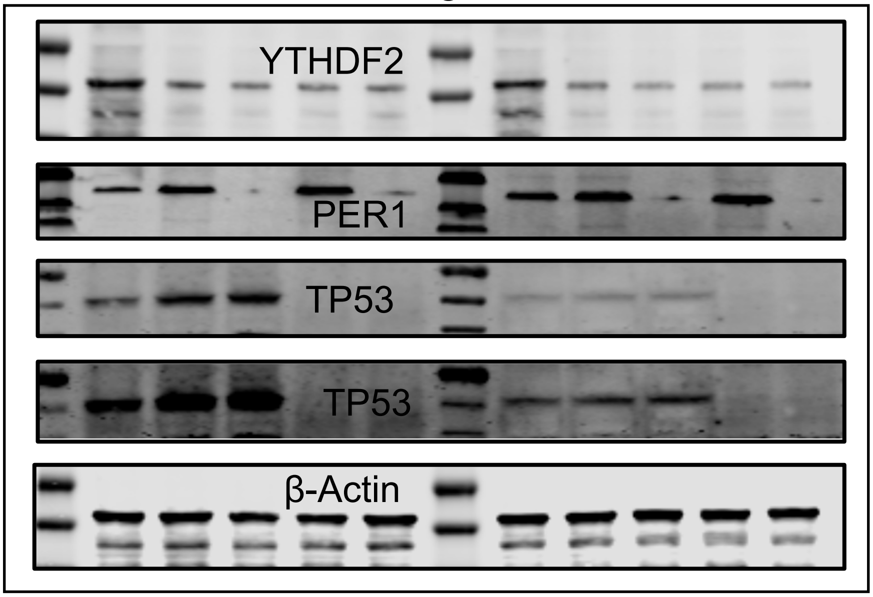

FigS5A

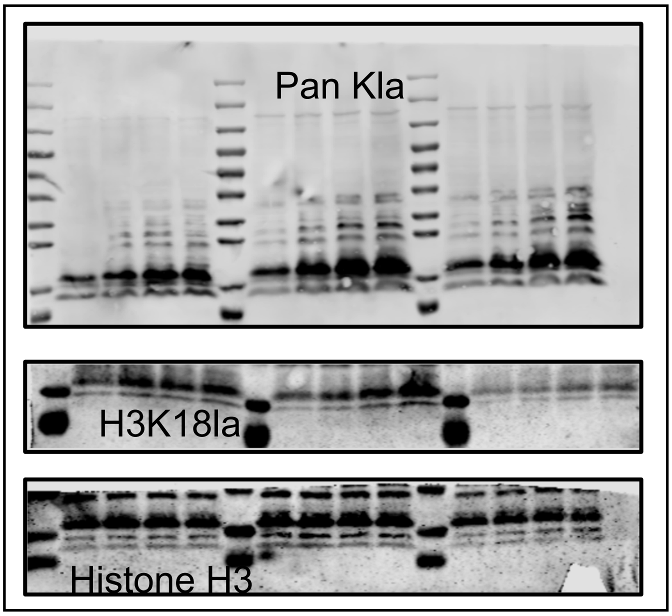

FigS5B

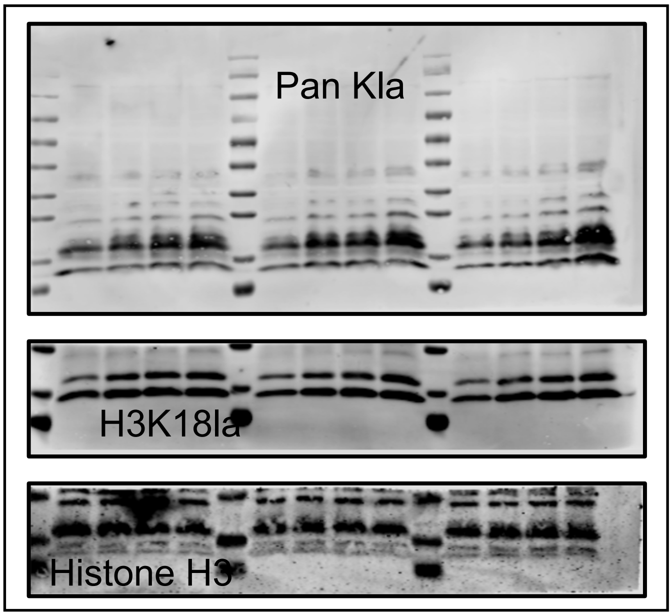

FigS6A

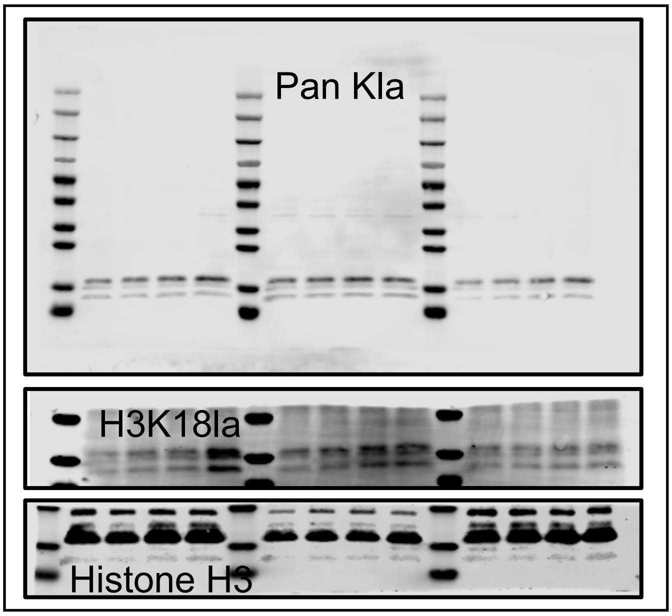

FigS7C

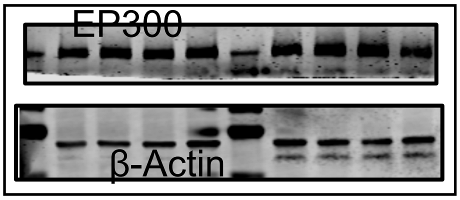

FigS7J

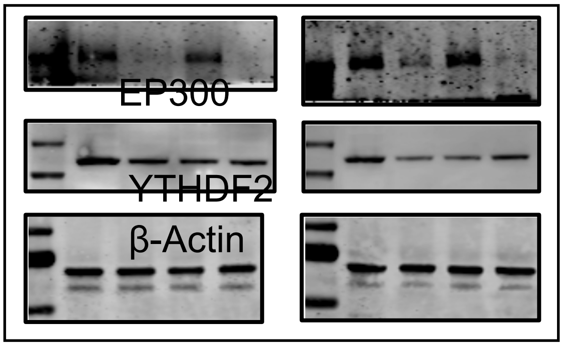

FigS11C

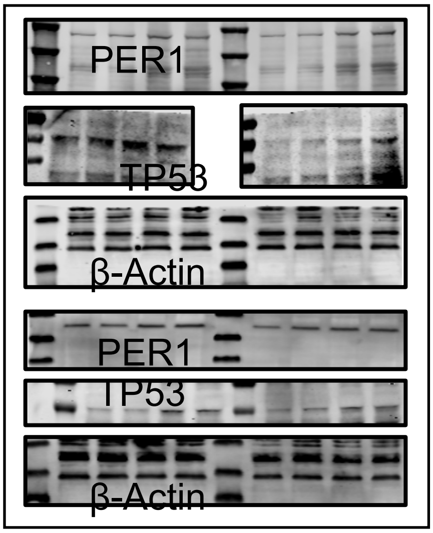

Supplement: Supplementary file 4 — Additional file 4. Uncropped western blotting analysis. [file 13059_2021_2308_MOESM4_ESM.pdf]
