# Supplementary material for: Histone lactylation drives oncogenesis by facilitating m6A reader protein YTHDF2 expression in ocular melanoma
Source: Genome Biol. 2021 Mar 16;22:85. doi: 10.1186/s13059-021-02308-z (PMC7962360; doi:10.1186/s13059-021-02308-z)
Supplement: Supplementary file 5 — Additional file 5. Review history. [file 13059_2021_2308_MOESM5_ESM.docx]

Review History

**First round of review**

**Reviewer 1**

**Are you able to assess all statistics in the manuscript, including the appropriateness of statistical tests used?** There are no statistics in the manuscript.

**Comments to author:**

In this manuscript, Yu et al. reported a potential role of histone lactylation in tumorigenesis. Specifically, the authors examined Ocular melanoma because this tumor type is highly glycolytic and produce large amounts of lactate. They found histone lactylation levels are higher in Ocular melanoma samples compared to normal melanocyte, and histone lactylation level is associated with aggressiveness of cancer. Blocking lactate production and histone lactylation using glycolysis inhibitors efficiently inhibits tumorigenesis. By ChIP-seq and RNA-seq analysis, the authors identified YTHDF2 as a target of histone lactylation. YTHDF2 can partially rescue the tumor suppression by glycolysis inhibitors. Mechanistically, YTHDF2 is a reader of m6A on mRNAs and down regulate tumor suppressor genes PER1 and TP53. Aggressive lactate production is a hallmark of cancer metabolism. Histone lactylation has recently been identified but not been studied in cancer. This study is therefore interesting and important for the understanding the metabolic-epigenetic link in cancer.

Specific comments:

Figure 1A-1D. Histone acetylation is also reported to be regulated by glycolysis. Is histone acetylation level differently regulated in Ocular melanoma versus normal melanocyte?

Figure 1C. How was the sample size determined by "low lactylation (n=26)" and "high lactylation (n=30)"? Why are not all 82 tumor samples (in Figure 1B) analyzed in this survival analysis?

Figure 1D, 1L. No details were described for the analysis, such as the sample size in each group, how lactylation levels were measured, etc.

Figure 2. 2-DG and Oxamate are non-specific glycolysis inhibitors, which may have other effects independent of inhibiting lactate production and lactylation. Can restoring histone lactylation levels by, for example, adding back lactate rescue the phenotype and gene expression? Does LDHA silencing corroborates glycolysis inhibitors' effects?

Figure 3G-H. In the ChIP-qPCR assay, p300 occupancy in YTHDF2 promoter is decreased by glycolysis inhibitor. Is it gene specific or because p300 is generally down regulated by glycolysis inhibitor? Is histone acetylation level decreased in these promoters since p300 is an acetyltransferase?

Figure 4G. How was the sample size determined by "low YTHDF2 (n=28)" and "high YTHDF2 (n=27)"? Why are not all 82 tumor samples analyzed for this survival analysis?

Figure 4G, 4H. No details were described for the analysis, such as sample size in each group, how was YTHDF2 levels are measured, etc.

Figure 7. The authors only show two targets from their MeRIP-seq/meCLIP-seq data. Are there other direct targets in addition to PER1 and TP53? Why did the authors pick these two targets?

Figure 7. Are expression levels of PER1 and TP53 lower in Ocular melanoma samples compared to normal melanocytes? Do glycolysis inhibitors increase PER1 and TP53 levels? Does lactate decrease PER1 and TP53 levels?

Other comments:

1. Introduction. Most of the examples in the Introduction section are not necessary; they are either not comprehensive or not closely relevant to the subject of this study.

2. Discussion. The first paragraph in the Discussion section is redundant with Introduction. I would suggest the authors to remove it.

**Reviewer 2**

**Are you able to assess all statistics in the manuscript, including the appropriateness of statistical tests used?** No, I do not feel adequately qualified to assess the statistics.

**Comments to author:**

In the manuscript entitled, "Histone lactylation drives oncogenesis by facilitating m6A reader protein YTHDF2 expression in ocular melanoma" by Yu et al. studies the role of Histone lactylation and its role in ocular melanoma. The authors provide data that elevated Histone lactylation is associated with a poor prognosis in ocular melanoma. Inhibition of glycolysis using 2DG and oxamate reduced overall histone lactylation levels and reduced survival, migration capacity, and tumor formation of ocular melanoma cell lines. Next, they performed H3K18la ChIP-seq which identified enrichment of H3K18la at promoter regions of metabolic genes and tumor-related genes. By overlapping with published database, the authors identified YTHDF2 as one of the targets. The authors further showed that the binding of H3K18la to YTHDF2 is reduced upon treatment of glycolysis inhibitors, which is associated with reduced YTHDF2 protein abundance. Overexpression of YTHDF2 partially rescued the growth inhibitory effect of glycolysis inhibitors in two cell lines. Similar to histone lactylation, the authors found that YTHDF2 expression was upregulated in ocular melanoma and high YTHDF2 was associated with poor prognosis. Knockdown of YTHDF2 reduced survival, and migration capacity of ocular melanoma cell lines. The authors showed two tumor suppressors Per1 and p53 as YTHDF2 targets, as their levels negatively correlate in database. They provided evidence that per1 and p53 transcripts were m6A modified and were preferentially bound by YTHDF2. Overall the manuscript contains a large set of interesting data that is of interest. However, there are several general weaknesses in the manuscript that include:

1) the causal link between histone lactylation and ocular melanoma oncogenesis. 2) mechanism behind the histone lactylation's control on YTHDF2 expression. 3) The mode of action of YTHDF2 and the link to m6A

1. There is pretty nice correlation between histone lactylation levels to ocular melanoma oncogenesis. However, the causal link remains to be further substantiated. The authors use the two glycolysis inhibitors as perturbation of glycolysis and lactate synthesis pathways that mimic the loss of function on histone lactylation levels. Could the authors include at least another inhibitor such as sodium dichloroacetate (DCA) and rotenone in the system or even better a genetic knockdown of LDHA? One would expect DCA also impair oncogenesis as it inhibits lactate production by inhibiting PDH, while rotenone mimics gain of function in histone lactylation as it inhibits mitochondria respiration and drives cells towards glycolysis. See Zhang et al Nature 2020, PMID: 31645732

2. What is the effect of inhibiting histone lactylation in normal cells? Is there any change in histone lactylation levels in PIG1 cells treated with the glycolysis inhibitors? In fig 2F looks like there is some inhibitory effect in PIG1 cells upon 2DG treatment but not oxamate.

3. Normal cells have a low lactylation level compared to cancer cells. What will happen if you increase histone lactylation in normal cells?

4. The mechanisms on how H3K18la controls YTHDF2 expression remain unclear. Does treatment of glycolysis inhibitors affect the nascent transcription of YTHDF2 transcript? Does it affect the mRNA stability of the transcript? Is the regulation mediated through p300? Would knockdown of p300 reverse the effect of glycolysis inhibitors in YTHDF2 expression?

5. The authors showed a nice correlation between YTHDF2 levels and ocular melanoma oncogenesis. As YTHDF2 is a m6A reader, is there a difference in m6A level between normal and ocular melanoma cell lines? Does lactylation inhibition affect m6A level in cells?

6. The mode of action of YTHDF2 in ocular melanoma is rather immature. It seems to me that the authors picked Per1 and p53 as YTHDF2 targets based on the mere fact that the two are tumor suppressors and their level negatively correlate with YTHDF2 in the TCGA database. There are thousands of genes that show negative and positive correlation, including the two oncogenes (NRAS and BRAF) that positively correlate with YTHDF2 expression. Thus, it needs more support on why the picked two are targets, for example by rescue experiments, or by a comprehensive knockdown RNAseq with overlap to YTHDF2 RIP-seq to identify direct targets. Further, there is no data on how YTHDF2 affects the mRNA stability of the two transcripts. Does depletion of m6A by METTL3 knockdown mimic the effect of YTHDF2 knockdown in ocular melanoma cells? What is the protein abundance of PER1 and p53 upon YTHDF2 knockdown?

**Authors Response**

**Point-by-point responses to the reviewers’ comments:**

Reviewer 1:

In this manuscript, Yu et al. reported a potential role of histone lactylation in tumorigenesis. Specifically, the authors examined Ocular melanoma because this tumor type is highly glycolytic and produce large amounts of lactate. They found histone lactylation levels are higher in Ocular melanoma samples compared to normal melanocyte, and histone lactylation level is associated with aggressiveness of cancer. Blocking lactate production and histone lactylation using glycolysis inhibitors efficiently inhibits tumorigenesis. By ChIP-seq and RNA-seq analysis, the authors identified YTHDF2 as a target of histone lactylation. YTHDF2 can partially rescue the tumor suppression by glycolysis inhibitors. Mechanistically, YTHDF2 is a reader of m6A on mRNAs and down regulate tumor suppressor genes PER1 and TP53. Aggressive lactate production is a hallmark of cancer metabolism. Histone lactylation has recently been identified but not been studied in cancer. This study is therefore interesting and important for the understanding the metabolic-epigenetic link in cancer.

Specific comments:
*Q1. Figure 1A-1D. Histone acetylation is also reported to be regulated by glycolysis. Is histone acetylation level differently regulated in Ocular melanoma versus normal melanocyte?*

**Response: Thank you, we have further compared global histone acetylation level between ocular melanoma cells (OCM1, MEL290, 92.1, OMM1, MUM2B, CRMM1, CRMM2 and CM2005.1) and normal melanocyte (PIG1). The result indicated histone acetylation levels were generally higher in ocular melanoma cell lines, compared to control cells (Rebuttal Figure 1), which could be contributed by elevated level of glycolysis. We would like to further demonstrate the oncogenic role of histone acetylation in our successive study.**

***Rebuttal figure 1: Histone acetylation level in ocular melanoma and control melanocytes***

*Q2. Figure 1C. How was the sample size determined by "low lactylation (n=26)" and "high lactylation (n=30)"? Why are not all 82 tumor samples (in Figure 1B) analyzed in this survival analysis?*

**Response: We defined relative lactylation level of the samples (determined by luminance intensity, please refer to Q3) higher than 0.87 as “high lactylation”, while those lower than 0.87 as “low lactylation”. We have added the definition in the legend of Figure 1C.**

**In addition, the prognostic information was available in 56 patients, while others were lost during follow-up or ruled out by unqualified immunofluorescence images (in Figure 1B). We have specified this information in the method section (Page. 19, para. 1).**

*Q3. Figure 1D, 1L. No details were described for the analysis, such as the sample size in each group, how lactylation levels were measured, etc.*

**Response: In Figure 1D, there were 3 ocular melanoma samples in T1 stage, 36 in T2 stage, 40 in T3 stage, and 3 in T4 stage. And in Figure 1L, there were 3 ocular melanoma samples in T1 stage, 35 in T2 stage, 41 in T3 stage, and 3 in T4 stage. We have specified this information in the figure legend of Figure 1D, 1L.**

**Relative lactylation level is determined by comparing the fluorescence intensity of the target antibody with that of DAPI [target fluorescence intensity (488nm absorbance)/DAPI intensity (405nm absorbance)]. We have specified this information in the method section (Page. 20, para. 2).**

*Q4. Figure 2. 2-DG and Oxamate are non-specific glycolysis inhibitors, which may have other effects independent of inhibiting lactate production and lactylation. Can restoring histone lactylation levels by, for example, adding back lactate rescue the phenotype and gene expression? Does LDHA silencing corroborates glycolysis inhibitors' effects?*

**Response: Thanks for your comments. Since 2-DG and oxamate may harbor other effects independent of inhibiting lactate production and lactylation, we therefore silenced two important enzymes for lactate production, LDHA and LDHB, for inhibiting** **histone lactylation (Figure 2A, rebuttal Figure 2A). We found either LDHA-deficient (Figure 2F, lane 2) or LDHB-deficient (Figure 2F, lane 3) ocular melanoma cells did not present with remarkable reduction of global histone lactylation. However, simultaneous silencing LDHA and LDHB (Figure 2F, lane 4) significantly impaired histone lactylation (Rebuttal Figure 2B), and in parallel, triggered a significant inhibition of cellular proliferation (Figure 2G-J) and migration (Figure 2K-L, rebuttal Figure 2C-L).**

**In addition, adding back sodium lactate (Nala) into LDHA and LDHB simultaneously silenced cells successfully increased histone lactylation levels (Figure 2F, lane 5) and partially restored cellular proliferation (Figure 2G-J) and migration (Figure 2K-L, rebuttal Figure 2C-H). We have added all these data in Rebuttal Figure 2 and Page. 8, para. 1.**

***Rebuttal figure 2:*** ***Simultaneous silencing LDHA and LDHB reduced lactyation levels and inhibited ocular melanoma tumorigenesis* A**. Schematic diagram of histone lactylation inhibition methods target. **B**. Lactyation and H3K18la levels were detected in OCM1 and CRMM1 cells after LDHA and LDHB silencing by Western blot. **C and D**. Proliferation of OCM1 (C) and CRMM1 (D) cells after LDHA and LDHB silencing was analyzed using CCK8 assay. **p<0.01, ****p<0.0001. **E**. Tumor growth of OCM1 and CRMM1 cells after LDHA and LDHB silencing was evaluated by colony formation assay. **F**. Statistical analysis of the colony formation assay performed using OCM1 and CRMM1 cells after LDHA and LDHB silencing. All of the experiments were performed in triplicate, and relative colony numbers are shown as means ± SD. *p<0.05, ***p<0.001. **G**. The migratory ability of OCM1 and CRMM1 cells after LDHA and LDHB silencing was evaluated by transwell assay. **H**. Statistical analysis of cells in the transwell assay performed using OCM1 and CRMM1 cells after LDHA and LDHB silencing. All of the experiments were performed in triplicate, and relative cell numbers are shown as means ± SD. *p<0.05, ***p<0.001.

*Q5. Figure 3G-H. In the ChIP-qPCR assay, p300 occupancy in YTHDF2 promoter is decreased by glycolysis inhibitor. Is it gene specific or because p300 is generally down regulated by glycolysis inhibitor? Is histone acetylation level decreased in these promoters since p300 is an acetyltransferase?*

**Response: Thanks for your critical comment. We found global p300 protein expression remained unchanged after being treated with glycolysis inhibitors (Figure S7C). In addition, H3K27ac level was slightly decreased at the YTHDF2 promoter under this condition (Figure S7D-E). Notably, CBP/EP300 is an indicative writer protein for diversified histone acylations, including histone acetylation, lactylation and crotonylation. Whether other EP300-guided histone modifications participated in YTHDF2 transcriptional regulation requires further explorations. We have revised relevant text in Page. 10, para. 1 and added more discussion in Page. 16, para. 1.**

*Q6. Figure 4G. How was the sample size determined by "low YTHDF2 (n=28)" and "high YTHDF2 (n=27)"? Why are not all 82 tumor samples analyzed for this survival analysis?*

**Response: We defined relative YTHDF2 expression level of the samples (determined by luminance intensity, please refer to Q3) higher than 0.72 as “high YTHDF2”, while those lower than 0.72 as “low YTHDF2”. We have added the definition in the figure legend of Figure 4G.**

**In addition, the prognostic information was available in 55 patients, while others were lost during follow-up or ruled out by unqualified immunofluorescence images (in Figure 4G). We have specific this information in the method section (Page. 19, para. 1).**

*Q7. Figure 4G, 4H. No details were described for the analysis, such as sample size in each group, how was YTHDF2 levels are measured, etc.*

**Response: In Figure 4H, there were 2 ocular melanoma samples in T1 stage, 36 in T2 stage, 42 in T3 stage, and 2 in T4 stage. We have specified this information in the figure legend of Figure 4H.**

**Relative YTHDF2 level is determined by comparing the fluorescence intensity of the target antibody with that of DAPI [target fluorescence intensity (488nm absorbance)/DAPI intensity (405nm absorbance)]. We have specified this information in the method section (Page. 20, para. 2).**

*Q8. Figure 7. The authors only show two targets from their MeRIP-seq/meCLIP-seq data. Are there other direct targets in addition to PER1 and TP53? Why did the authors pick these two targets?*

**Response: Thanks for your comments. First of all, GO analysis of YTHDF2 negatively correlated genes in TCGA database showed that they were enriched in DNA repair and cell cycle pathways** **(Figure S9A). Then,** **by combining MeRIP-seq data (GEO accession number: GSE137675) with RNA-seq data of cells treated with glycolysis inhibitors or not (GEO accession number: GSE156675), we identified that 13 genes with m^6^A peaks in 3’UTR were significantly increased in cells treated with glycolysis inhibitors and showed negative correlation (|R|>0.4, p<0.05) with YTHDF2 expression in TCGA database (Figure 7A, rebuttal Figure 3A). Further real-time PCR assays showed that 2 of the genes (PER1 and TP53) were remarkably upregulated (Fold Change>5, p<0.05) in both two tumor cell lines after being treated with glycolysis inhibitors (Figure S9D-E, rebuttal Figure 3B-C). In addition, PER1 has been reported to be downregulated in an m6A-YTHDF2-dependent manner[1]. We therefore picked PER1 and TP53 as potential targets of YTHDF2. We have revised the text in Page. 12, para. 2.**

***Rebuttal figure 3: PER1 and TP53 may serve as the key candidate gene of YTHDF2***

**A**. Bioinformatics analysis filtered PER1 and TP53 may serve as potential downstream target of YTHDF2. **B and C**. RT-PCR were performed to test potential downstream targets of YTHDF2 obtained by bioinformatics analysis in OCM1 (B) and CRMM1 (C) cells.

*Q9. Figure 7. Are expression levels of PER1 and TP53 lower in Ocular melanoma samples compared to normal melanocytes? Do glycolysis inhibitors increase PER1 and TP53 levels? Does lactate decrease PER1 and TP53 levels?*

**Response: As suggested, we have performed immunofluorescence staining of PER1 and TP53 in our ocular melanoma tissue chip, the data confirmed that PER1 and TP53 levels were significantly lower in ocular melanoma tissue samples comparing to normal melanocyte tissue samples (Figure S12A-C, rebuttal Figure 4A-C). We have revised text in Page. 13, para. 2.**

**In addition, we observed PER1 and TP53 were downregulated by histone lactylation inducer (Nala) (Figure 7K, rebuttal Figure 4D) and upregulated by histone lactylation inhibitors (2-DG and oxamate) (Figure S11C, rebuttal Figure 4E). We have also revised the text in Page. 13, para. 2.**

***Rebuttal figure 4: PER1 and TP53 may serve as the key candidate gene of YTHDF2***

**A**. PER1 and TP53 expression were visualized by immunofluorescence staining in normal and tumor tissues. **B**. Statistical results of PER1 expression in normal and tumor tissues. Unpaired t test, ** p<0.01. **C**. Statistical results of TP53 expression in normal and tumor tissues. Unpaired t test, ** p<0.01. **D**. Expression of PER1 and TP53 was detected in OCM1 and CRMM1 cells treated with different concentrations of Nala for 24 h by Western blot. **E**. Expression of PER1 and TP53 was detected in OCM1 and CRMM1 cells treated with different concentrations of 2-DG and oxamate for 24 h by Western blot.

Other comments:
*Q1. Introduction. Most of the examples in the Introduction section are not necessary; they are either not comprehensive or not closely relevant to the subject of this study.*

**Response: Thanks, we have added recent studies** **related to histone lactylation in Page. 4, para. 2.**

*Q2. Discussion. The first paragraph in the Discussion section is redundant with Introduction. I would suggest the authors to remove it.*

**Response: Thanks for your suggestion. We have removed duplicated text in Page. 15, para. 1.**

**-Reviewer 2**

In the manuscript entitled, "Histone lactylation drives oncogenesis by facilitating m6A reader protein YTHDF2 expression in ocular melanoma" by Yu et al. studies the role of Histone lactylation and its role in ocular melanoma. The authors provide data that elevated Histone lactylation is associated with a poor prognosis in ocular melanoma. Inhibition of glycolysis using 2DG and oxamate reduced overall histone lactylation levels and reduced survival, migration capacity, and tumor formation of ocular melanoma cell lines. Next, they performed H3K18la ChIP-seq which identified enrichment of H3K18la at promoter regions of metabolic genes and tumor-related genes. By overlapping with published database, the authors identified YTHDF2 as one of the targets. The authors further showed that the binding of H3K18la to YTHDF2 is reduced upon treatment of glycolysis inhibitors, which is associated with reduced YTHDF2 protein abundance. Overexpression of YTHDF2 partially rescued the growth inhibitory effect of glycolysis inhibitors in two cell lines. Similar to histone lactylation, the authors found that YTHDF2 expression was upregulated in ocular melanoma and high YTHDF2 was associated with poor prognosis. Knockdown of YTHDF2 reduced survival, and migration capacity of ocular melanoma cell lines. The authors showed two tumor suppressors Per1 and p53 as YTHDF2 targets, as their levels negatively correlate in database. They provided evidence that per1 and p53 transcripts were m6A modified and were preferentially bound by YTHDF2. Overall the manuscript contains a large set of interesting data that is of interest. However, there are several general weaknesses in the manuscript that include: 1) the causal link between histone lactylation and ocular melanoma oncogenesis. 2) mechanism behind the histone lactylation's control on YTHDF2 expression. 3) The mode of action of YTHDF2 and the link to m6A

*Q1. There is pretty nice correlation between histone lactylation levels to ocular melanoma oncogenesis. However, the causal link remains to be further substantiated. The authors use the two glycolysis inhibitors as perturbation of glycolysis and lactate synthesis pathways that mimic the loss of function on histone lactylation levels. Could the authors include at least another inhibitor such as sodium dichloroacetate (DCA) and rotenone in the system or even better a genetic knockdown of LDHA? One would expect DCA also impair oncogenesis as it inhibits lactate production by inhibiting PDH, while rotenone mimics gain of function in histone lactylation as it inhibits mitochondria respiration and drives cells towards glycolysis. See Zhang et al Nature 2020, PMID: 31645732*

**Response: Since 2-DG and oxamate may harbor other effects independent of inhibiting lactate production and lactylation, we therefore silenced two important enzymes for lactase production, LDHA and LDHB, for inhibiting** **histone lactylation. we found either LDHA-deficient (Figure 2F, lane 2) or LDHB-deficient (Figure 2F, lane 3) ocular melanoma cells did not present with remarkable reduction of global histone lactylation. However, simultaneous silencing LDHA and LDHB (Figure 2F, lane 4) significantly impaired histone lactylation, and in parallel, triggered a significant inhibition of cellular proliferation (Figure 2G-J) and migration (Figure 2K-L). Please refer to Review1-Q4.**

**As for gain of function assays, sodium lactate, glucose, and rotenone were used to elevate histone lactylations in ocular melanoma cells (Figure S5A-B, rebuttal Figure 5A-B). However, we did not observe significant changes in cell growth (Figure S5C-D, rebuttal Figure 5C-D) or colony formation ability (Figure S5E-H, rebuttal Figure 5E-H) of ocular melanoma cells after upregulating histone lactylation levels. Taken together, upregulation of histone lactylation could not further promote tumor progression in ocular melanoma cells. It could be resulted from that elevated histone lactylation levels had reached a saturation in ocular melanoma, which had been sufficient for tumorigenesis. We have revised texts in Page. 8, para. 1.**

***Rebuttal figure 5: Effects of histone lactyation elevation on ocular melanoma cells* A and B.** Lactyation and H3K18la levels were detected in OCM1 (A) and CRMM1 (B) cells treated with different concentrations of Nala, glucose, or rotenone by Western blot. **C and D**. Proliferation of OCM1 (C) and CRMM1 (D) cells treated with different concentrations of Nala, glucose, or rotenone was analyzed using CCK8 assay. **E and G**. Tumor growth of OCM1 (E) and CRMM1 (G) cells treated with different concentrations of Nala, glucose, or rotenone was evaluated by colony formation assay. **F and H**. Statistical analysis of the colony formation assay performed using OCM1 (F) and CRMM1 (H) cells treated with different concentrations of Nala, glucose, or rotenone. All experiments were performed in triplicate, and relative colony numbers are shown as means ± SD.

*Q2. What is the effect of inhibiting histone lactylation in normal cells? Is there any change in histone lactylation levels in PIG1 cells treated with the glycolysis inhibitors? In fig 2F looks like there is some inhibitory effect in PIG1 cells upon 2DG treatment but not oxamate.*

**Response: Western blot assays showed that there’s a slight decrease in histone lactylation levels in PIG1 cells treated with the glycolysis inhibitors (Rebuttal Figure 6), which could be caused by low levels of histone lactylation in PIG1 cells. Moreover, additions of glycolysis inhibitors did not reduce histone lactylation levels as much as that of tumor cells and caused little inhibitory effect in PIG1 cells. In addition, 2-DG, a glucose analog, affects not only glycolysis but also tricarboxylic acid cycle, so that’s probably why there is some inhibitory effect in PIG1 cells as well.**

***Rebuttal figure 6: Histone lactylation levels in*** ***PIG1 cells treated with glycolysis inhibitors***

*Q3. Normal cells have a low lactylation level compared to cancer cells. What will happen if you increase histone lactylation in normal cells?*

**Response: As suggested, sodium lactate, glucose, and rotenone were used to elevate histone lactylations in normal melanocytes (Figure S6A, rebuttal Figure 7A), however, we did not observe significant changes in cell growth (Figure S6B, rebuttal Figure 7B) or colony formation ability (Figure S6C-D, rebuttal Figure 7C-D). These data indicated that upregulating histone lactylation alone could not transform normal cells into tumors. Although elevated histone lactylation was required for tumor progression of ocular melanoma, multi-factor cooperation is warranted during tumorigenesis. We have revised text in Page. 8, para. 1.**

***Rebuttal figure 7: Effects of histone lactyation elevation on control melanocyte* A**. Lactyation and H3K18la levels were detected in PIG1 cells treated with different concentrations of Nala, glucose, or rotenone by Western blot. **B**. Proliferation of PIG1 cells treated with different concentrations of Nala, glucose, or rotenone was analyzed using CCK8 assay. **C**. Tumor growth of PIG1 cells treated with different concentrations of Nala, glucose, or rotenone was evaluated by colony formation assay. **D**. Statistical analysis of the colony formation assay performed using PIG1 cells treated with different concentrations of Nala, glucose, or rotenone. All of the experiments were performed in triplicate, and relative colony numbers are shown as means ± SD.

*Q4. The mechanisms on how H3K18la controls YTHDF2 expression remain unclear. Does treatment of glycolysis inhibitors affect the nascent transcription of YTHDF2 transcript? Does it affect the mRNA stability of the transcript? Is the regulation mediated through p300? Would knockdown of p300 reverse the effect of glycolysis inhibitors in YTHDF2 expression?*

**Response: Thank you for your comments. We have performed mRNA stability assays and it demonstrated that glycolysis inhibitors had no effect on the stability of YTHDF2 mRNA (Figure S7H-I). In addition, either p300 silencing or glycolysis inhibitors remarkably attenuated histone lactylation in YTHDF2 promoter and in parallel, a significant reduction of YTHDF2 expression was observed. Moreover, we noticed YTHDF2 expression remain unchanged by simultaneously silencing p300 and glycolysis inhibition, compared with p300-deficent or glycolysis inhibited group (Figure S7J). These results suggested that glycolysis inhibition suppressed YTHDF2 expression mainly through regulating EP300 docking in its promoter region. We have** **revised text in Page. 10, para. 1.**

*Q5. The authors showed a nice correlation between YTHDF2 levels and ocular melanoma oncogenesis. As YTHDF2 is a m6A reader, is there a difference in m6A level between normal and ocular melanoma cell lines? Does lactylation inhibition affect m6A level in cells?*

**Response: Thank you for your comments, we have performed m6A dot plot compared global m6A level between ocular melanoma cells (OCM1, MEL290, 92.1, OMM1, MUM2B, CRMM1, CRMM2 and CM2005.1) and normal melanocyte (PIG1). The result indicated global m6A levels were generally lower in ocular melanoma cell lines, compared to normal control cells (Rebuttal Figure 8A), which was consistent with our previous report[2]. And lactylation inhibition did not affect global m6A level in cells (Rebuttal Figure 8B).**

***Revised figure 8: m6A level in normal and ocular melanoma cells and in ocular melanoma cells treated with glycolysis inhibitors***

*Q6. The mode of action of YTHDF2 in ocular melanoma is rather immature. It seems to me that the authors picked Per1 and p53 as YTHDF2 targets based on the mere fact that the two are tumor suppressors and their level negatively correlate with YTHDF2 in the TCGA database. There are thousands of genes that show negative and positive correlation, including the two oncogenes (NRAS and BRAF) that positively correlate with YTHDF2 expression. Thus, it needs more support on why the picked two are targets, for example by rescue experiments, or by a comprehensive knockdown RNAseq with overlap to YTHDF2 RIP-seq to identify direct targets. Further, there is no data on how YTHDF2 affects the mRNA stability of the two transcripts. Does depletion of m6A by METTL3 knockdown mimic the effect of YTHDF2 knockdown in ocular melanoma cells? What is the protein abundance of PER1 and p53 upon YTHDF2 knockdown?*

**Response: Thank you for your thoughtful suggestion. We have carried out the** **rescue experiments by silencing PER1 and TP53 in YTHDF2 knockdown ocular melanoma cells. As a result, silencing PER1 and TP53 partially restored cellular proliferation (Figure 8B-E) and migration (Figure 8F-G) in YTHDF2-deficient ocular melanoma cells. The results further suggested that PER1 and TP53 were** **targets of YTHDF2. In addition, we have investigated the PER1 and TP53 mRNA stability alterations in YTHDF2-deficient cells. It showed that YTHDF2 silencing significantly increased mRNA stability of PER1 and TP53 (Figure S11D-E). As expected, the protein abundance of PER1 and p53 were remarkably upregulated upon YTHDF2 knockdown** **(Figure 7J). We have revised text in Page. 13, para. 2.**

**In addition, the role of METTL3 is different from YTHDF2 depletion in ocular melanoma. We have previously revealed METTL3 function as a tumor suppressor genes in ocular melanoma[2], and METTL3 has been contributed to enhanced response to anti-PD-1 treatment in melanoma patients. However, we found YTHDF2 was upregulated and function as an oncogenic driver in ocular melanoma. It should be noted that METTL3 is the major writer of m6A RNA methylation which catalyzed wide varieties of RNA targets. Meanwhile, YTHDF2 functions as a reader protein which are regulator for stability and translational efficacy of m6A modified RNA. They may share common m6A modified targets and overlapping functions, however, the bio-behavior and molecular function may differ when encountering specific RNA candidates.**

**References**

1.	Guo X, Li K, Jiang W, Hu Y, Xiao W, Huang Y, Feng Y, Pan Q, Wan R: **RNA demethylase ALKBH5 prevents pancreatic cancer progression by posttranscriptional activation of PER1 in an m6A-YTHDF2-dependent manner.** *Mol Cancer* 2020, **19:**91.

2.	Jia R, Chai P, Wang S, Sun B, Xu Y, Yang Y, Ge S, Jia R, Yang Y-G, Fan X: **m6A modification suppresses ocular melanoma through modulating HINT2 mRNA translation.** *Molecular Cancer* 2019, **18:**161.

**Second round of review**

**Reviewer 1**

The authors have adequately addressed my concerns.

**Reviewer 2**

The authors have responded to all the comments.
